# MicroRNA-101-3p Suppresses Cancer Cell Growth by Inhibiting the USP47-Induced Deubiquitination of RPL11

**DOI:** 10.3390/cancers14040964

**Published:** 2022-02-15

**Authors:** Jinyoung Park, Moonsoo Cho, Jinhong Cho, Eunice EunKyeong Kim, Eun Joo Song

**Affiliations:** 1 Molecular Recognition Research Center, Korea Institute of Science and Technology, Hwarangno 14-gil 5, Seongbuk-gu, Seoul 027921, Korea; lansur92@ewha.ac.kr; 2 Graduate School of Pharmaceutical Sciences, College of Pharmacy, Ewha Womans University, Seoul 03760, Korea; chomaro@ewha.ac.kr; 3 Biomedical Research Institute, Korea Institute of Science and Technology, Hwarangno 14-gil 5, Seongbuk-gu, Seoul 02792, Korea; eunice@kist.re.kr

**Keywords:** MiR-101-3p, USP47, RPL11, p53, cancer cell growth

## Abstract

**Simple Summary:**

An abnormal expression of microRNA is commonly observed in cancer. Since a single miRNA can target numerous genes, it is important to understand the exact mechanism for the regulation of cancer growth by miRNAs. Here, we show that miR-101-3p, which is downregulated in several cancers, regulates RPL11 ubiquitination by targeting USP47, thereby controlling p53 levels by affecting the localization of RPL11 and its interaction with MDM2. Our results provide a novel mechanism for the inhibition of cancer cell growth by miR-101-3p, and suggest that miR-101-3p could be a potential target as an anticancer agent.

**Abstract:**

MicroRNAs (miRNAs) are a class of small non-coding RNA molecules that regulate a countless number of genes in the cell, and the aberrant expression of miRNA can lead to cancer. Here, we demonstrate that miR-101-3p regulates the RPL11–MDM2–p53 pathway by targeting ubiquitin-specific peptidase 47 (USP47), consequently inhibiting cancer cell proliferation. We confirm that miR-101-3p directly binds to the 3′-UTR region of the USP47 gene and inhibits USP47 expression. In addition, the overexpression of miR-101-3p suppresses cell proliferation in a p53-dependent manner. MiR-101-3p promotes interaction between RPL11 and MDM2 by inducing the translocation of RPL11 from the nucleolus to the nucleoplasm, thus preventing the MDM2-mediated proteasomal degradation of p53. However, these phenomena are restored by the overexpression of USP47, but not by its catalytically inactive form. Indeed, miR-101-3p regulates RPL11 localization and its interaction with MDM2 by inhibiting the USP47-induced deubiquitination of RPL11. Finally, the expression of miR-101-3p is downregulated in lung cancer patients, and the patients with low miR-101-3p expression exhibit a lower survival rate, indicating that miR-101-3p is associated with tumorigenesis. Together, our findings suggest that miR-101-3p functions as a tumor suppressor by targeting USP47 and could be a potential therapeutic target for cancers.

## 1. Introduction

MicroRNAs (miRNAs) are a class of small single-stranded RNA molecules containing approximately 21 to 25 nucleotides with no protein-coding sequences [1,2]. Mature miRNA has a seed sequence that binds to the 3′ UTR region of a target mRNA sequence, thereby regulating the levels and functions of a target gene [3,4]. Studies have shown that miRNAs regulate cellular processes, including cell proliferation, apoptosis, and development, and that the dysregulation of miRNAs is associated with many types of diseases, including cancers [5]. MiRNAs can act as both oncogenes and tumor suppressors. For example, miR-15a and miR-16-1 are mutated or decreased in expression in leukemia, which has been found to be associated with tumorigenesis, and they induce apoptosis by targeting an anti-apoptotic protein, Bcl-2, in many solid malignancies [6,7,8]. High miR-21/miR-29a expression results in the progression of metastasis by directly binding to Toll-like receptor 7 (TLR7) or TLR8, and promoting TLR-mediated prometastatic inflammatory responses [9].

Many miRNAs are involved in the p53-related responses of various cancers. For example, MiR-34a regulates p53 expression by reducing SIRT1 levels, promoting cell cycle arrest and apoptosis [10]. In addition, miR-192, miR-194, and miR-215 bind directly to mouse double minute 2 homolog (MDM2) mRNA and inhibit its expression, thereby maintaining p53 stability by protecting MDM2-mediated proteasomal degradation in multiple myeloma [11]. MiR-101-3p is also known to induce G2 arrest and apoptosis in a p53-dependent manner [12]. 

The dysregulation of ribosome biogenesis by ribosomal stress, such as hypoxia, starvation, UV, and chemotherapeutic agents, leads to p53 activation, which is a major cellular response to cell cycle arrest or apoptosis [13]. In response to ribosomal stress, many ribosomal proteins (RPs) interact with MDM2 and inhibit the MDM2-mediated proteasomal degradation of p53 [14,15,16,17,18]. RPL11 plays a pivotal role in p53 stabilization and activation by ribosomal stress. While other RPs are expeditiously synthesized and degraded during the stress, RPL11 is resistant to proteasomal degradation, and free RPL11 translocates from the nucleolus to the nucleoplasm, where it binds to MDM2 [14]. Since disruption of the RPL11–MDM2 complex accelerates tumorigenesis through the degradation of p53, the RPL11–MDM2–p53 pathway plays a critical role in interrupting cancer progression.

Here, we show that miR-101-3p enhances the RPL11-induced defense mechanism against abnormal cell proliferation by targeting ubiquitin-specific peptidase 47 (USP47) in cancers. The suppression of cell proliferation by miR-101-3p is rescued by an overexpression of USP47. In addition, USP47 binds to and deubiquitinates RPL11, and USP47-induced RPL11 deubiquitination is inhibited by miR-101-3p. Furthermore, miR-101-3p affects the RPL11 functions that maintain p53 stability by inducing interaction with MDM2 and re-localization from the nucleolus to the nucleoplasm. The effect of miR-101-3p on the interaction with MDM2 and the localization of RPL11 is eliminated by the overexpression of USP47 but not by the overexpression of the catalytically inactive form of USP47, suggesting that the deubiquitination of RPL11 by USP47 is required to inhibit RPL11 functions. Finally, miR-101-3p expression levels are downregulated in lung cancer patients, and the miR-101-3p mimic suppresses cell proliferation in a p53-dependent manner. From these results, we conclude that miR-101-3p regulates ribosomal protein functions by inhibiting USP47 expression, thus controlling p53 activity.

## 2. Materials and Methods

### 2.1. Cell Culture and Drugs

All the cell lines were purchased from Korea Cell Line Bank (KCLB, Seoul, Korea). The A549, H460, H1650, H1299, U2OS, and HCT116 cells were maintained in RPMI-1640, and the HEK293T cells were maintained in Dulbecco’s Modified Eagle medium (DMEM) supplemented with 10% fetal bovine serum and 1% penicillin–streptomycin (GenDEPOT, Katy, TX, USA). The cells were incubated in a humidified incubator with 5% CO_2_ at 37 °C. All the cell lines used in this study were authenticated by KCLB and confirmed to be free of mycoplasma contamination prior to use. MG132 (AG Scientific, San Diego, CA, USA) and cycloheximide (CHX) (Sigma-Aldrich, Burlington, MA, USA) were dissolved in DMSO. All drugs were used with the indicated concentration in the legend.

### 2.2. Transient Transfection

For plasmid transfection, 2 M CaCl_2_ and 2X HBS buffer (50 mM HEPES, 10 mM KCl, 12 mM Glucose, 280 mM NaCl, and 1.5 mM Na_2_HPO_4_ at pH 7.5) were used in the HEK293T cells, and Effectene (Qiagen, Hilden, Germany) was used in other cells following the manufacturer’s instructions. For the siRNA and miRNA transfection, Lipofectamine^TM^ 2000 or 3000 (Invitrogen, Waltham, MA, USA) was used following the manufacturer’s instructions. 

### 2.3. Plasmids, siRNAs, and miRNA

pFlag-CMV^TM^-2 vector, pFlag-CMV^TM^-2-USP47, and pFlag-CMV^TM^-2-USP47^C109S^ (catalytically inactive mutant) were kindly provided by CH Chung. pCMV-MDM2 was kindly given by SH Baek. Ubiquitin and RPL11 were cloned into pCS2-His or -HA, vectors for expression in mammalian cells. Mimics of negative control and miR-101-3p were purchased from Genolution (Seoul, Korea). Control siRNA and siRNAs targeting USP47 (USP47i) were synthesized from Bioneer (Seoul, Korea). The miRNA mimic and siRNA sequences were as follows: miR-101-3p mimic sense 5′-UACAGUACUGUGAUAACUGAA-3′ and antisense 5′-UUCAGUUAUCACAGUACUGUA-3′. The *USP47* siRNA sequences were as follows: #1; 5′-TGAAAAGGGATGTGCAAAA-3′ and #2; 5’-AAGCTACTCCTACTCATCTATTT-3’.

### 2.4. Western Blot Analysis

Western blot analyses on 10–50 μg protein extracts from the cells were performed as described earlier. The cells were lysed using a protein lysis buffer (20 mM Tris-HCl pH 7.5, 150 mM NaCl, 1 mM EDTA, 1 mM EGTA, 1% Triton X-100, 2.5 mM Na_4_P_2_O_7_, 50 mM NaF, 5 mM β-glycerophosphate, 1 mM Na_3_VO_4_) containing protease inhibitor (Roche, Basel, Switzerland). The lysates were quantified by a Micro BCA^TM^ Protein Assay kit (Thermo Fisher Scientific, Waltham, MA, USA) based on the standard curve using BSA. The following antibodies were used for immunoblot analysis: rabbit anti-USP47 (A301-048A, Bethyl Laboratories, Montgomery, TX, USA), rabbit anti-RPL11 (ab79352, Abcam), mouse anti-p53 (sc-126, Santa Cruz, Dallas, TX, USA), mouse anti-MDM2 (PA5-11353, Invitrogen), anti-p21 (2947S, Cell signaling Technology, Danvers, MA, USA), mouse anti-XIAP (sc-55551, Santa Cruz), mouse anti-caspase 3 (sc-271028, Santa Cruz), mouse anti-HSP90α/β (sc-13119, Santa Cruz), rabbit anti-β-actin (LF-PA0207, Ab frontier, Seoul, Korea), mouse anti-HA (sc-7392, Santa Cruz), and mouse anti-Flag (F1804, Sigma-Aldrich). The band intensity was quantified using image J and normalized by an indicated loading control protein. Original images of the western blot can be found in Supplementary File S1.

### 2.5. Immunoprecipitation

HA- or Flag-tagged plasmids were transfected in HEK293T cells. The cells were collected and lysed with a protein lysis buffer. Approximately 3–5 mg of lysates were incubated with monoclonal anti-HA agarose beads (Sigma-Aldrich), or monoclonal anti-Flag magnetic beads (Sigma-Aldrich) for 5 h at 4 °C. For fully endogenous immunoprecipitation, approximately 5mg of lysates were incubated with rabbit IgG (02-6102, Thermo Fisher Scientific), or rabbit anti-RPL11 for 4 h at 4 °C. After incubation, protein A agarose beads (GenDEPOT) were added and incubated again o/n at 4 °C. The immunocomplexes were washed five times with a lysis buffer and eluted and boiled in 6X SDS buffer for 5 min at 97 °C. All samples were detected by Western blot analysis using the indicated antibodies, and 5% of the samples were used to identify immunoprecipitation efficiency.

### 2.6. Ni-NTA Pull-Down Assay

His-ubiquitin and plasmids or miR-101-3p mimic were co-transfected in HEK293T cells. After treatment with 10 μM MG132 for 4 h, the cells were lysed with urea lysis buffer (8 M urea, 0.3 M NaCl, 50 mM Na_2_HPO_4_, 50 mM Tris-HCl, 1 mM PMSF, 10 mM imidazole) and sonicated for 4 min. The same amounts of lysates were incubated with Ni-NTA agarose (Qiagen) for 6 h at 4 °C. Beads were washed five times with urea washing buffer (8 M urea, 0.3 M NaCl, 50 mM Na_2_HPO_4_, 50 mM Tris-HCl, 1 mM PMSF, 20 mM imidazole) and eluted in 6X SDS buffer at 97 °C for 5 min. The samples were detected by Western blot analysis.

### 2.7. Quantitative Real-Time PCR (qRT-PCR)

Total RNA was extracted using Trizol reagent (Invitrogen) according to the manufacturer’s instructions. To detect the USP47 gene expression levels, cDNA was synthesized using the ReverTraAce^®^ qPCR RT Master Mix Kit (Toyobo, Osaka, Japan). For miRNA detection, cDNA was synthesized using the Mir-X miRNA First-Stranded Synthesis Kit (Clontech, Mountain View, CA, USA). qRT-PCR was performed using the SYBR^®^ Green Realtime PCR Master Mix (Toyobo). The mRNA levels were normalized to β-actin expression, and the miRNA levels were normalized to U6 expression. The relative expression levels were calculated using the delta-delta Ct method (ddCt). The following primers were used: *USP47* forward 5′-ATGGAAGAATCCTGGCACTG-3′, *USP47* reverse 5′-CTGGAAGGGATCCAACTTCA-3′; *TP53* forward 5′-CCTCAGCATCTTATCCGAGTGG-3′, *TP53* reverse 5′-TGGATGGTGGTACAGTCAGAGC-3′; *CDKN1A* forward 5′-CTGGAGACTCTCAGGGTCGAAA-3′, *CDKN1A* reverse 5′-GATTAGGGCTTCCTCTTGGAGAA-3′; *PMAIP1* forward 5′-GTGCCCTTGGAAACGGAAGA-3′, *PMAIP1* reverse 5′-CCAGCCGCCCAGTCTAATCA-3′; *BBC3* forward 5′-GGGCCCAGACTGTGAATCCT-3′, *BBC3* reverse 5′-ACTTGCTCTCTCTAAACCTATGCA-3′; *β-actin* forward 5′-CTCTTCCAGCCTTCCTTCCT-3′, *β-actin* reverse 5′-AGCACTGTGTTGGCGTACAG-3′; and miR-101-3p forward 5′- TACAGTACTGTGATAACTGAA -3′, the mRQ 3′ primer was provided using the Mir-X miRNA First-Stranded Synthesis Kit (Clontech).

### 2.8. Luciferase Assay

The miR-101-3p binding sequence of USP47 3′-UTR was identified from the TargetScan database program. This sequence was synthesized (Cosmogenetech, Seoul, Korea) and cloned into the pmiR-GLO luciferase reporter vector (Promega, Madison, WI, USA). The sequences of these vectors were verified by DNA sequencing. The MiR-101-3p mimic and luciferase reporter plasmid containing wild-type or mutant USP47 3′-UTR was co-transfected in A549 cells. The luciferase assays were performed using the Dual-Glo^®^ Luciferase Assay System (Promega) on a SpectraMax^®^ M3 (Molecular Devices, San Jose, CA, USA). All data were normalized to Renilla luciferase activity. The following sequences of USP47 3′-UTR were used: wild-type USP47 3′-UTR forward, 5′-AAACTAGCGGCCGCTAGTGTGCACTATAGTCAAATGTACTGTAT-3′; wild-type USP47 3′-UTR reverse, 5′-CTAGATACAGTACATTTGACTATAGTGCACACTAGCGGCCGCTAGTTT-3′; mutant USP47 3′-UTR forward, 5′-AAACTAGCGGCCGCTAGTGTGCACTATAGTCAAATTGCAGTGAT-3′; and mutant USP47 3′-UTR reverse, 5′-CTAGATCACTGCAATTTGACTATAGTGCACACTAGCGGCCGCTAGTTT-3′.

### 2.9. WST-1 Assay

To measure the cell viability, 1 × 10^3^ U2OS cells and 3x 103 A549, H460, and H1650 cells were seeded into 6 wells of 96-well plates per group 24 h before transfection. After transfection with 10 nM miR-101-3p mimic or Flag-tagged USP47 plasmid alone, or together, a WST-1 assay was performed at an indicated time using EZ-cytox (DoGenBio, Seoul, Korea) following the manufacturer’s instructions. Day 0 means just before transfection 24 h after cell seeding. The absorbance was measured on a microplate absorbance reader (Bio-Rad, Hercules, CA, USA) (equipped at Ewha Drug Development Research Core Center) at a wavelength of 450 nm. WST-1 assays were performed independently in triplicate.

### 2.10. Colony Formation Assay

The cells were transfected with 50 nM miR-101-3p mimic or Flag-USP47 plasmid alone, or together. The cells were resuspended with trypsin-EDTA 48 h after transfection, and 500 cells were seeded in 6-well plates. After incubation for 10 days, the cells were fixed with 100% ice-cold methanol for 20 min and stained with crystal violet (Sigma-Aldrich) for 1 h.

### 2.11. Immunofluorescence

The U2OS cells were co-transfected with HA-tagged RPL11 plasmid and miR-101-3p mimic using Lipofectamine 2000^TM^ reagent. After incubation for 48 h, the cells were fixed with 100% ice-cold methanol for 10 min, and then permeabilized in PBS containing 0.25% Triton X-100 for 10 min. After washing, the cells were stained with anti-HA (sc-7392, Santa Cruz) overnight at 4 °C, followed by Alexa Fluor 488-conjugated goat anti-mouse (Molecular Probes, Eugene, OR, USA) for 1 h at room temperature. The nucleolus was stained with anti-fibrillarin (ab5821, Abcam), and DNA was detected by DAPI (Sigma-Aldrich). The fluorescence response HA-RPL11 and fibrillarin staining cells were visualized using the Nikon ECLIPS Ti-U fluorescence microscope (Nikon, Tokyo, Japan) and analyzed using NIS-Elements imaging software (Nikon, Tokyo, Japan, ver 4.20).

### 2.12. Statistical Analysis

The results are shown as mean ± standard deviation of at least three independent experiments, unless otherwise indicated in the figure legends. All statistical analyses were obtained using Student’s *t*-tests. *p*-values < 0.05 were considered statistically significant.

## 3. Results

### 3.1. MiR-101-3p Suppresses Cancer Cell Growth in a p53-Dependent Manner

**Figure 1:**
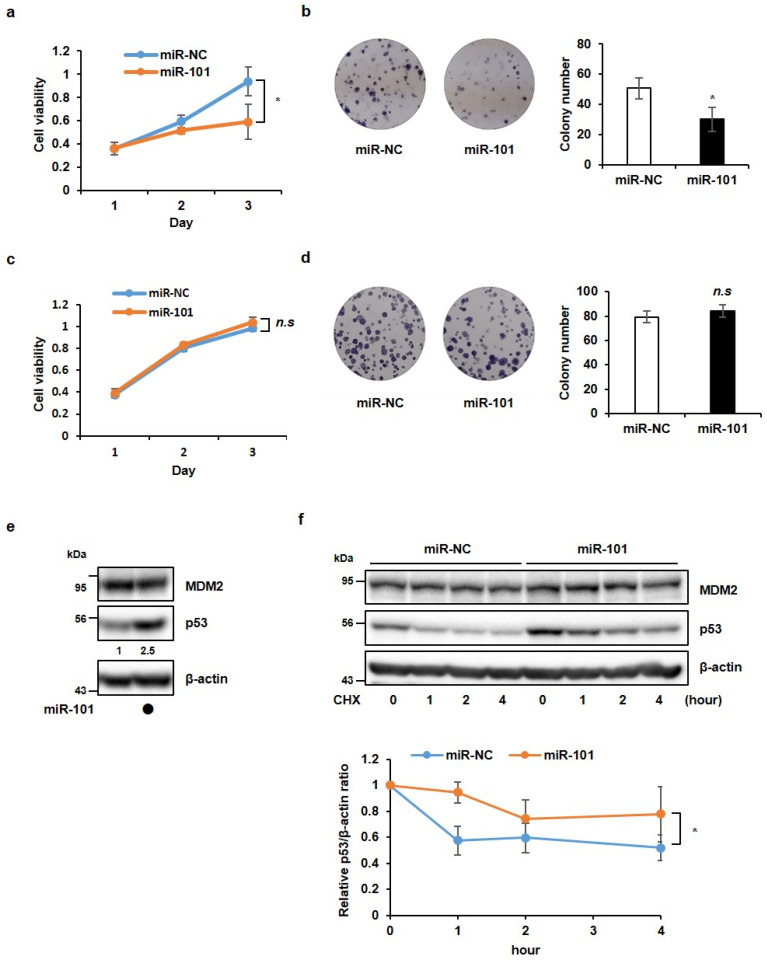
MiR-101-3p inhibits cancer cell growth in a p53-dependent manner. (**a**,**b**) A549 cells were transfected with a miR-101-3p mimic. (**a**) Cell viability was measured at the time indicated by a WST-1 assay. (**b**) For the colony formation assay, cells were cultured for 10 days and stained with crystal violet in a 6-well plate. (**c**,**d**) H1299 cells were transfected with a miR-101-3p mimic. (**c**) Cell viability was measured at the time indicated by a WST-1 assay. (**d**) For the colony formation assay, cells were cultured for 10 days and stained with crystal violet in a 6-well plate. (**e**) Western blot analysis was performed to detect p53 and MDM2 protein levels in lysates from A549 cells transfected with the miR-101-3p mimic. β-actin was used as a loading control. (**f**) U2OS cells transfected with the miR-101-3p mimic were treated with CHX (100 μg/mL) at the times indicated. Western blot analysis was conducted to detect p53 protein levels. Quantification of p53 levels was carried out considering the amount of β-actin protein in each case. The data in parts (**a**–**d**) and (**f**) represent the mean ± SD (* *p* < 0.05, *t*-test). *n.s.*, non-specific.

Previous studies have shown that miR-101-3p suppresses tumorigenesis and is downregulated in diverse types of cancer [19,20,21,22,23]. However, the molecular mechanism of miR-101-3p as a tumor suppressor in cancer has not been widely studied. To identify the function of miR-101-3p in cancer, we examined the alteration of cancer cell phenotypes by the overexpression of miR-101-3p using a miR-101-3p mimic. Before testing the effects of miR-101-3p, we confirmed the transfection efficiency using qRT-PCR analysis. As shown in Figure S1a, the miR-101-3p expression was upregulated by transfection with the miR-101-3p mimic. First, cell viability was assessed at 1, 2, and 3 days after transfection with the miR-101-3p mimic in A549 lung cancer cells. Using the WST-1 cell viability assay, we observed that miR-101-3p significantly inhibited cell viability compared with the negative control (Figure 1a). Next, we measured the effect of miR-101-3p on cancer cell growth using a colony formation assay. In A549 cells, the miR-101-3p mimic exhibited less cell growth than the controls (Figure 1b). These results demonstrated that miR-101-3p negatively controls cancer cell growth.

Based on a recent report indicating that miR-101-3p forms a molecular circuit with p53 and that this circuit functions as an intrinsic tumor-suppressor network in response to ribosomal stress [22], we attempted to verify whether the effect of miR-101-3p on cell proliferation is associated with p53. H1299 lung cancer cells, which do not express p53, were transfected with a miR-101-3p mimic, and a WST-1 assay and colony formation assay were performed. As shown in Figure 1c,d, we were not able to observe any difference in the miR-101-3p-overexpressed H1299 cells compared with the control H1299 cells. We also examined whether miR-101-3p functions dependent on p53 or not using HCT116 colorectal cancer cells. Both the wild-type HCT116 cells (HCT116 (p53^+/+^)) and the p53-knockout HCT116 cells (HCT116 (p53^−/−^)) were transfected with the miR-101-3p mimic, and the WST-1 assay was performed. We observed that cell viability decreased with the overexpression of miR-101-3p in the HCT116 (p53^+/+^) cells, but not in the HCT116 (p53^−/−^) cells (Figure S2a,c). Additionally, the colony formation assay showed that the miR-101-3p mimic reduces cell growth in HCT116 (p53^+/+^) cells, but not in HCT116 (p53^−/−^) cells, compared with the negative control (Figure S2b,d). Therefore, we concluded that miR-101-3p suppresses cancer cell growth in a p53-dependent manner. These results prompted us to investigate how p53 affects the function of miR-101-3p in cancer cells. As shown in Figure 1e, the overexpression of miR-101-3p significantly increased the p53 protein levels. To further confirm whether miR-101-3p regulates the stability of p53 or not, we performed a protein degradation assay using CHX after the overexpression of the miR-101-3p mimic. At 0 min, the p53 protein levels were notably elevated by the miR-101-3p mimic relative to the negative control, and this elevated level was maintained until 4 h, whereas the p53 protein levels were almost degraded in the control at 4 h (Figure 1f). Taken together, these results suggest that miR-101-3p inhibits cell growth by maintaining the stability of p53. Indeed, miR-101-3p did not affect the levels or the stability of MDM2, an E3 ligase that promotes the proteasomal degradation of p53, which suggests that miR-101-3p regulates p53 stability regardless of MDM2 levels (Figure 1e,f).

### 3.2. MiR-101-3p Maintains the Stability of p53 by Reducing USP47 Levels

To elucidate the molecular mechanism underlying the miR-101-3p-mediated regulation of p53 stability, we searched for potential target genes by utilizing four different miRNA target prediction databases: miRWalk, TargetScan, miRanda, and RNA22. Among the hundreds of potential binding partners for miR-101-3p, we focused on a deubiquitinating enzyme, USP47, as a promising target.

Recently, Zhang et al. published that USP47 is a target gene of miR-101-3p, and our group have reported that USP47 regulates the levels of p53 via the inhibition of MDM2 activity indirectly [24,25]. Based on these papers, we hypothesized that USP47 regulates p53 protein levels, i.e., miR-101-3p stabilizes p53 via targeting USP47. To examine this possibility, we confirmed again whether the USP47 gene is a target of miR-101-3p ourselves. The qRT-PCR analysis revealed that miR-101-3p decreased the USP47 mRNA level (Figure S3a). Furthermore, the USP47 protein level was reduced when miR-101-3p was overexpressed (Figure S3b). In addition, by performing a pmiR-GLO dual-luciferase reporter assay using the cells transfected with wild-type or mutant pmiR-GLO vector and a miR-101-3p mimic, we confirmed that miR-101-3p directly targets the predicted sequence in the 3′-UTR region of USP47 mRNA. (Figure S3c,d). These results showed that miR-101-3p directly targets the USP47 gene by binding to the 3′-UTR region of USP47 mRNA.

**Figure 2:**
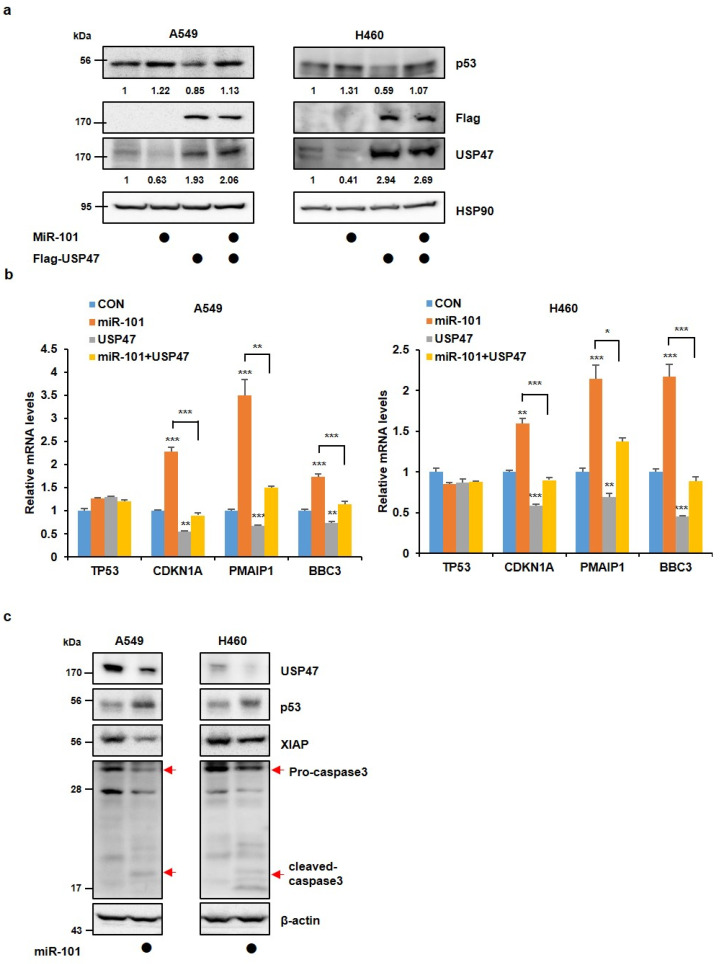
MiR-101-3p regulates p53 functions by targeting USP47. (**a**,**b**) A549 and H460 cells were transfected with a miR-101-3p mimic or Flag-USP47 alone, or together. (**a**) Western blot analysis was performed to detect the p53 protein level. (**b**) Q-PCR analysis was conducted to determine the relative expression levels of p53 and its target genes. (**c**) A549 and H460 cells were transfected with a miR-101-3p mimic. Western blot analysis was performed to detect apoptotic proteins. The data in a part (**b**) represent the mean ± SD (* *p* < 0.05, ** *p* < 0.005, *** *p* < 0.0005, *t*-test).

**Figure 3:**
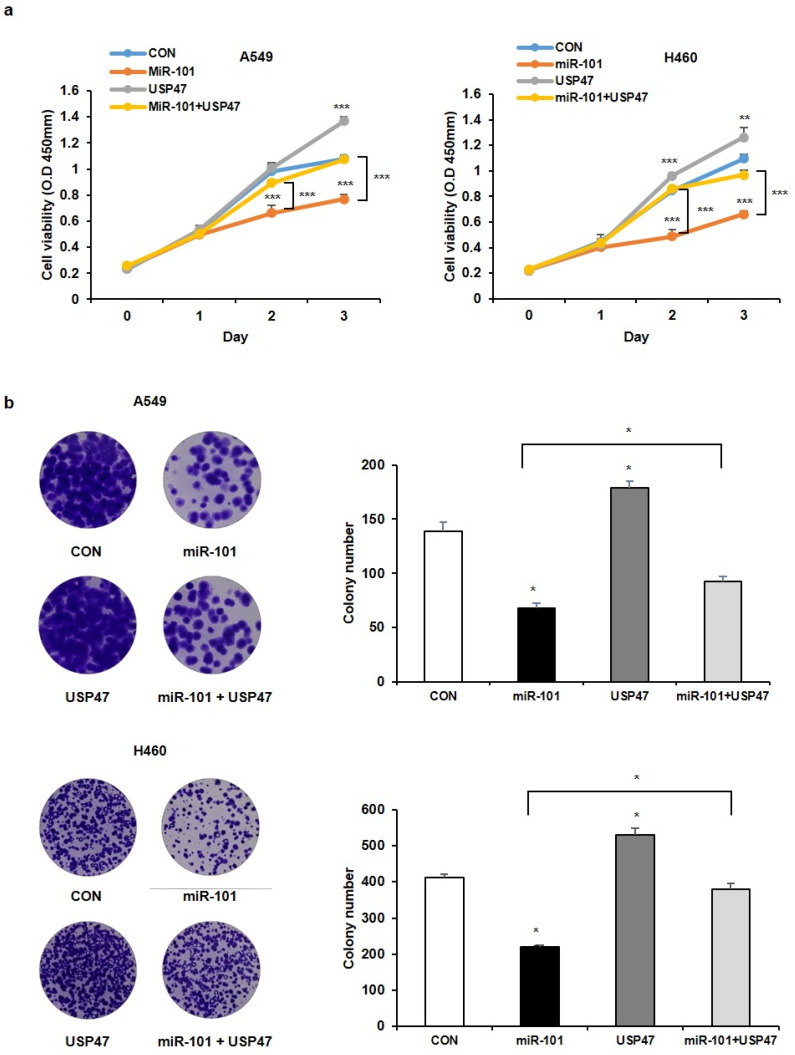
MiR-101-3p inhibits cancer cell growth by targeting USP47. (**a**,**b**) A549 and H460 cells were transfected with a miR-101-3p mimic or Flag-USP47 alone, or together. (**a**) Cell viability was measured at the time indicated by the WST-1 assay. (**b**) 15 days after transfection, the cells were stained with crystal violet in a 6-well plate. The number of colonies for each group was normalized to the control (CON). The data in all parts represent the mean ± SD (* *p* < 0.05, ** *p* < 0.005, *** *p* < 0.0005, *t*-test).

Next, to identify whether the miR-101-3p-induced upregulation of p53 levels might be carried out through USP47, we measured the p53 protein levels after transfection with miR-101-3p mimic alone, or together with USP47 plasmid. Ectopic miR-101-3p expression caused an increase in p53 protein levels, but this elevation was reduced by an overexpression of USP47, meaning that miR-101-3p regulates p53 levels through USP47 ablation (Figure 2a and Figure S4a). We also observed that the USP47 levels were decreased, while the p53 protein levels were maintained high after CHX treatment in miR-101-3p mimic-transfected cells compared with the control cells (Figure S5), as shown in Figure 1f. In addition, the expression of p53 target genes such as CDKN1A, PMAIP1, and BBC3 was increased by the miR-101-3p mimic, but when USP47 was overexpressed, the target gene levels were restored similar to that of the control group (Figure 2b and Figure S4b). In fact, the mRNA level of TP53 was not altered by miR-101-3p, suggesting that miR-101-3p controls the protein level of p53 by targeting USP47 and not directly targeting TP53. Increased stability or transcriptional activity of p53 induces apoptotic cell death [26,27]. To determine whether miR-101-3p regulates apoptosis through an increase in p53 stability, we measured the protein levels of XIAP and caspase 3, which are representative apoptosis markers. As a result, miR-101-3p decreased XIAP and pro-caspase 3, but increased a cleaved form of caspase 3, suggesting that miR-101-3p increased the apoptosis of cancer cells (Figure 2c and Figure S4c). As demonstrated earlier, miR-101-3p inhibits cancer cell growth by maintaining the p53 protein level. As the reduction in USP47 levels by miR-101-3p led to the accumulation of p53, we could surmise that the miR-101-3p-induced suppression of cancer cell growth was due to the reduction in USP47 levels. To address this point further, we monitored cell growth after transfection with the miR-101-3p mimic and USP47 plasmid by performing a WST-1 assay and a colony formation assay. MiR-101-3p reduced the cell viability and colony formation ability as expected, but the overexpression of USP47 reversed the reduction in cell viability and colony number (Figure 3a,b and Figure S4d,e). Therefore, we suggest that miR-101-3p regulates p53 levels by targeting USP47, which then leads to the reduced cancer cell numbers via apoptosis.

### 3.3. MiR-101-3p Regulates USP47-Induced Deubiquitination of RPL11

**Figure 4:**
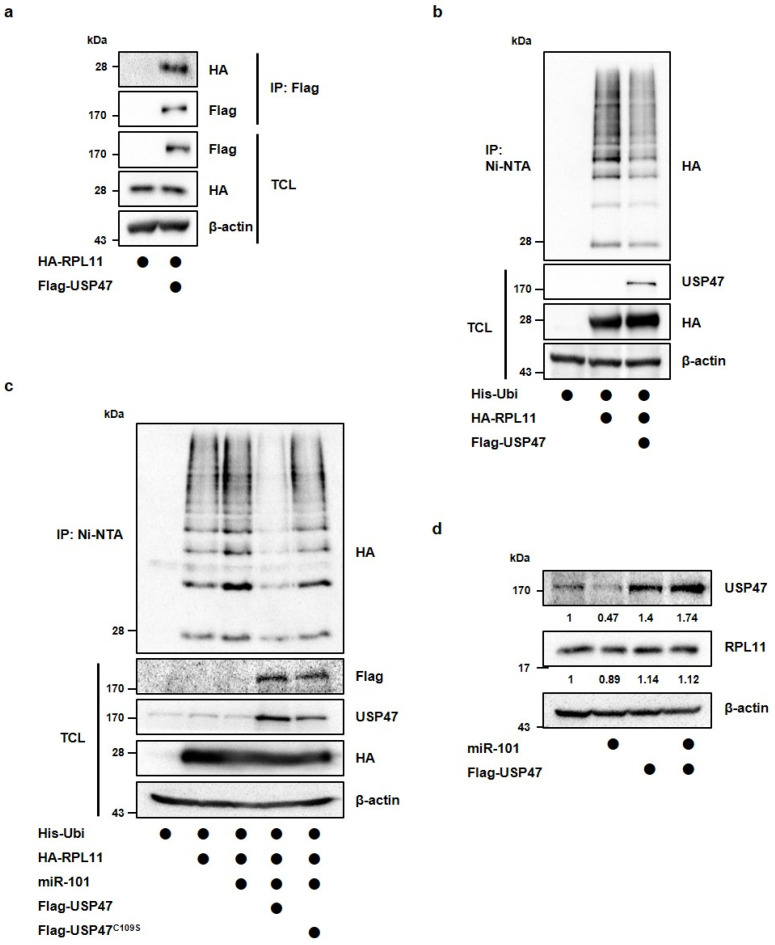
MiR-101-3p suppresses RPL11 deubiquitination by USP47. (**a**) HEK293T cells were transfected with HA-RPL11 alone or in combination with Flag-USP47. The interaction between Flag-USP47 and HA-RPL11 was detected by immunoblotting after immunoprecipitation with anti-Flag magnetic beads. (**b**) HEK293T cells were transfected with His-ubiquitin alone or together with HA-RPL11 and Flag-USP47, and then treated with proteasomal inhibitor MG132 (10 μM) for 4 h. RPL11 ubiquitination was observed using a Ni-NTA pulldown assay. (**c**) HEK293T cells were transfected with His-ubiquitin alone or together with HA-RPL11, miR-101-3p mimic, Flag-USP47, or Flag-USP47C109S, and then treated with proteasomal inhibitor MG132 (10 μM) for 4 h. RPL11 ubiquitination was observed using a Ni-NTA pulldown assay. (**d**) A594 cells were transfected with miR-101-3p mimic or Flag-USP47 alone, or together. Western blot analysis was performed to detect the USP47 and RPL11 protein levels. IP, immunoprecipitation; TCL, total cell lysates.

We investigated how miR-101-3p targets USP47 to regulate the stability of p53. Previously, we searched USP47 binding proteins using LC-MS analysis and found that USP47 interacts with several ribosomal proteins. Among them, RPL11 has been well established as the main regulator of MDM2 E3-ligase, and it participates in the p53 pathway upon ribosomal stress [14,28]. Therefore, we hypothesized that miR-101-3p may affect the RPL11 function by reducing the levels of USP47, which maintains p53 stability. To confirm this possibility, we first investigated the interaction between USP47 and RPL11. When we co-transfected with Flag-tagged USP47 and HA-tagged RPL11 in the cells, and we observed the binding of USP47 to RPL11 by immunoprecipitation with anti-Flag magnetic beads (Figure 4a). Next, we tested whether USP47 has catalytic activity on RPL11. By performing a Ni-NTA pull-down assay, we were able to observe that USP47 deubiquitinates RPL11 (Figure 4b). We also confirmed that PRL11 ubiquitination was reduced by the depletion of USP47 (Figure S6a,b). In contrast, RPL11 ubiquitination was elevated by the transfection of the miR-101-3p mimic, but the overexpression of wild-type USP47 (and not USP47^C109S^, a catalytically inactive form of USP47) reversed this ubiquitination (Figure 4c). The transfection efficiency of the miR-101-3p mimic into the HEK293T cells was confirmed by qRT-PCR analysis (Figure S1b,c). These results suggest that miR-101-3p regulates the ubiquitination of RPL11 by reducing the USP47 levels. Indeed, both miR-101-3p and USP47 did not alter the RPL11 levels (Figure 4d). Therefore, the modification of RPL11 by miR-101-3p or USP47 may regulate the cellular function of RPL11, not the RPL11 protein levels.

### 3.4. MiR-101-3p Regulates the Interaction between RPL11 and MDM2 by Translocation of RPL11

**Figure 5:**
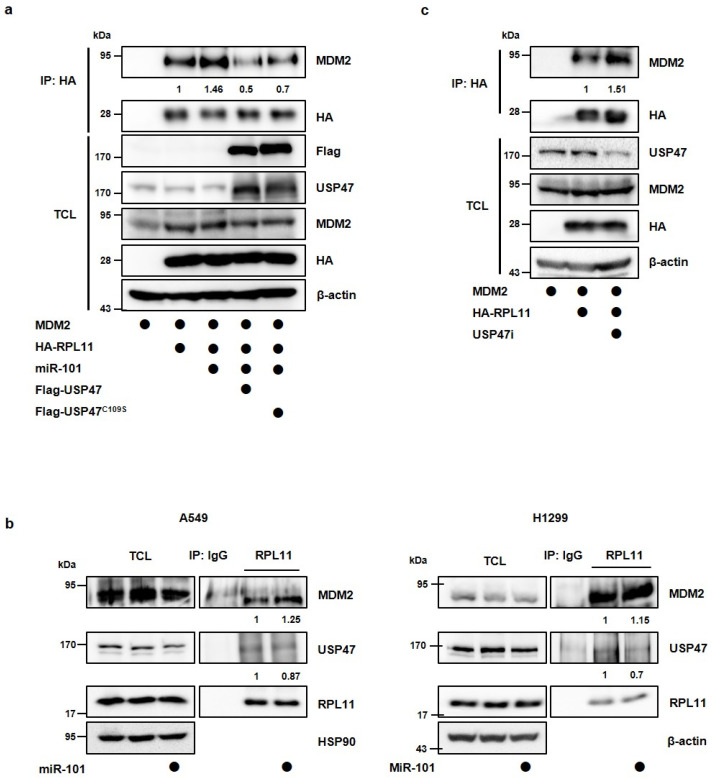
MiR-101-3p increases the interaction between RPL11 and MDM2. (**a**) HEK293T cells were co-transfected with MDM2, HA-RPL11, Flag-USP47, Flag-USP47^C109S^, and miR-101-3p mimic. The interaction between MDM2 and HA-RPL11 was detected by immunoblotting after immunoprecipitation with anti-HA agarose beads. The quantification of MDM2 levels in the IP samples was carried out considering the amount of HA in each case of IP samples. (**b**) A549 and H1299 cells were transfected with a miR-101-3p mimic. The interaction between endogenous RPL11 and MDM2, or RPL11 and USP47 was detected by immunoblotting after immunoprecipitation with anti-RPL11 antibody. Quantification of MDM2 or USP47 levels in the IP samples was carried out considering the amount of RPL11 in each case of IP samples. (**c**) HEK293T cells were transfected with siRNA targeting USP47 (USP47i) alone or together with MDM2 and HA-RPL11. The interaction between MDM2 and HA-RPL11 was detected by immunoblotting after immunoprecipitation with anti-HA agarose beads. Quantification of MDM2 levels in the IP samples was conducted considering the amount of HA in each case of IP samples.

**Figure 6:**
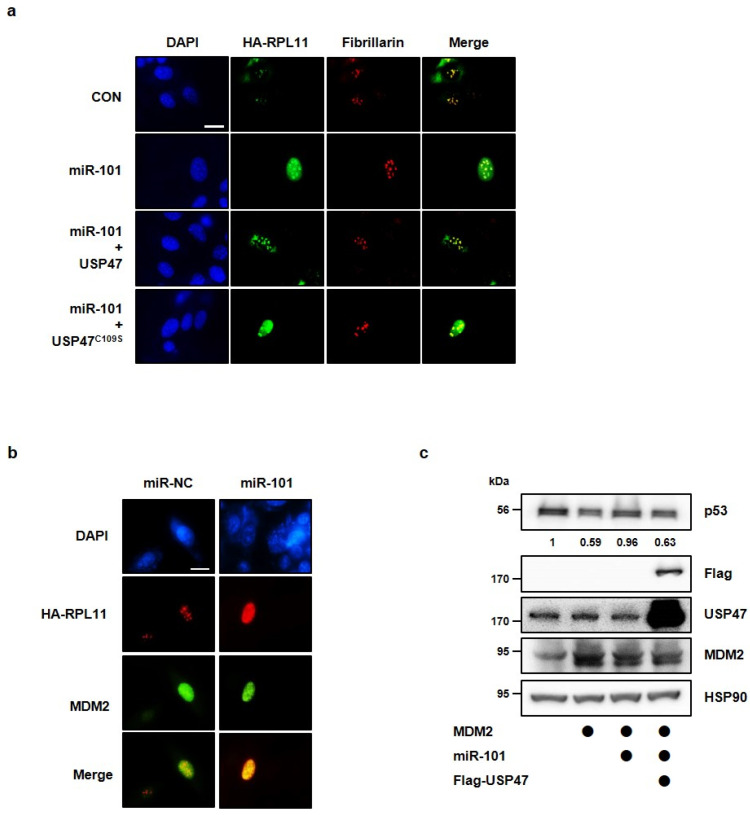
MiR-101-3p changes the localization of RPL11 by targeting USP47. (**a**) U2OS cells were transfected with HA-RPL11 alone or together with miR-101-3p mimic, Flag-USP47, or Flag-USP47C109S. Immunofluorescence staining was performed using an anti-HA antibody. The nucleoli were stained with an anti-fibrillarin antibody. Scale = 10 μm. (**b**) U2OS cells were transfected with HA-RPL11 alone or together with a miR-101-3p mimic. Immunofluorescence staining was performed using the anti-HA antibody and anti-MDM2-antibody. Scale = 10 μm. (**c**) A549 cells were transfected with MDM2 alone, or together with Flag-USP47 or a miR-101-3p mimic. Western blot analysis was performed to detect the p53 protein levels.

During ribosomal stress, ribosomal proteins, presumably including RPL11, are exported from the nucleolus to the nucleoplasm, where MDM2 is usually expressed, to interact with MDM2 E3-ligase inhibiting its activity [14]. Hence, we questioned whether miR-101-3p regulates the interaction between RPL11 and MDM2 through the regulation of USP47. To address this, we co-transfected HEK293T cells with HA-tagged RPL11, MDM2, miR-101-3p mimic, and Flag-tagged wild-type USP47 or USP47^C109S^. Interestingly, miR-101-3p increased the interaction of RPL11 and MDM2 compared with the control. Furthermore, the interaction promoted by miR-101-3p was rescued by the overexpression of USP47 but not by that of USP47^C109S^ (Figure 5a). Thus, we concluded that miR-101-3p regulates the interaction between MDM2 and RPL11 via the regulation of USP47 levels, and the catalytic activity of USP47 is required for the regulation of the interaction of RPL11 with MDM2. Consistent with this, RPL11 interacted with MDM2 or USP47 in physiological conditions, and the interaction between RPL11 and MDM2 was slightly increased by miR-101-3p (Figure 5b). We also investigated whether reduction of USP47 levels by siRNAs targeting USP47 shows the same results as overexpression of miR-101-3p. As expected, the USP47 knockdown increased the interaction between MDM2 and RPL11, as well as miR-101-3p (Figure 5c). Next, we examined whether miR-101-3p regulates the localization of RPL11. Using cells transfected with HA-tagged RPL11 alone, or together with the miR-101-3p mimic, we stained with anti-HA antibody and anti-fibrillarin antibody to detect RPL11 and the nucleolus, respectively. RPL11 was only detected in the nucleolus in the control cells. However, when transfected with the miR-101-3p mimic, RPL11 was also detected in the nucleoplasm (Figure 6a). This means that miR-101-3p affected the translocation of RPL11 from the nucleolus to the nucleoplasm. Moreover, RPL11 and MDM2 were co-localized in the cells transfected with the miR-101-3p mimic, suggesting that the shifting of RPL11 to the nucleoplasm by miR-101-3p allowed RPL11 to be located in the same region as MDM2, thereby increasing the interaction between RLP11 and MDM2 (Figure 6b).

To identify whether miR-101-3p induces the translocation of RPL11 from the nucleolus to the nucleoplasm by regulating USP47, we transfected with wild-type USP47 or USP47^C109S^ and observed the RPL11 localization in the cells. RPL11, which was transferred to the nucleoplasm by miR-101-3p, was re-localized to the nucleolus by the overexpression of USP47 but not by that of USP47^C109S^ (Figure 6a). In other words, the deubiquitination of RPL11 by USP47 affected the localization of RPL11. Finally, we looked at whether miR-101-3p affects the MDM2-induced proteasomal degradation of p53 by targeting USP47. After transfection with MDM2 alone, or together with the miR-101-3p mimic or USP47, we confirmed the p53 protein levels. As seen previously, the p53 levels were reduced by MDM2, but the miR-101-3p mimic reversed this reduction. At this time, the overexpression of USP47 decreased the p53 levels recovered by miR-101-3p (Figure 6c). 

### 3.5. Aberrant Expression of miR-101-3p and USP47 in Lung Cancer

**Figure 7:**
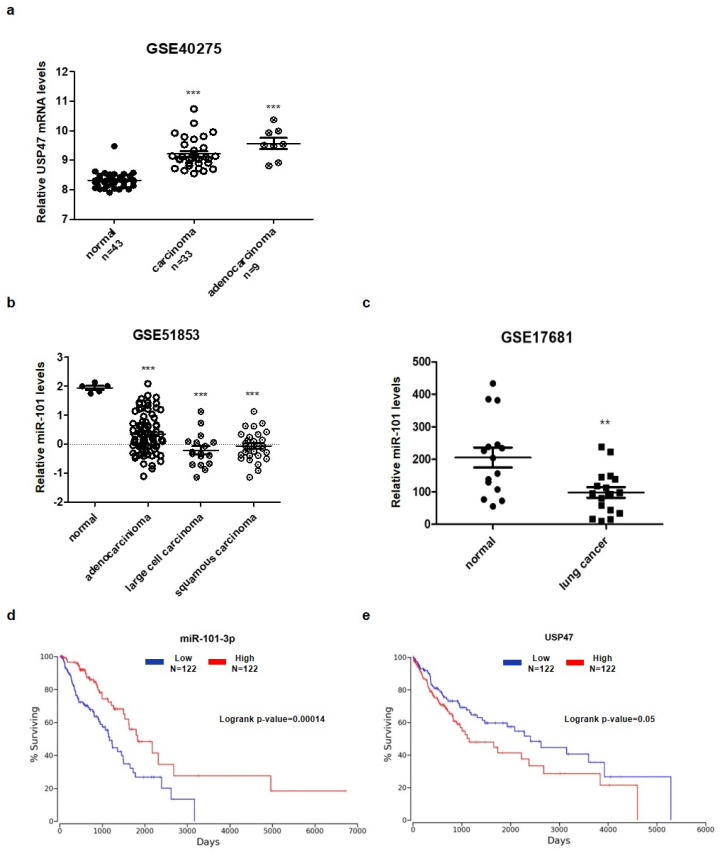
MiR-101-3p is downregulated in lung cancer. (**a**) USP47 expression level was analyzed in adjacent normal tissues (*n* = 43) and lung cancer tissues (*n* = 33, *n* = 9) (GSE40275). (**b**,**c**) MiR-101-3p expression levels were analyzed in: (**b**) adjacent normal tissues (*n* = 5) and lung cancer tissues (*n* = 119) (GSE51853); and (**c**) control (*n* = 19) and patient (*n* = 17) blood samples (GSE17681). (**d**,**e**) Kaplan–Meier analysis of survival rates according to: (**d**) miR-101-3p; or (**e**) USP47 expression in lung cancer patients performed using the TCGA database at the OncoLnc site. The data in parts (**a**–**c**) represent the mean ± SD (** *p* < 0.005, *** *p* < 0.001, *t*-test).

Previous studies have reported that USP47 positively regulates tumorigenesis [29,30,31,32]. In particular, a depletion in USP47 reduced tumor growth in xenograft models using A549 lung cancer cells [25]. We also confirmed that USP47 gene expression was higher in lung cancer tissues through the analysis of microarray data obtained from the National Center for Biotechnology Information’s Gene Expression Omnibus (GEO) database (accession number GSE40275) (Figure 7a). To investigate the expression pattern of miR-101-3p in lung cancer patients, we analyzed the expression levels of miR-101-3p using microarray data of tissues (76 adenocarcinomas, 14 large cell carcinomas, and 29 squamous cell carcinomas out of a total of 119 lung cancer specimens and 5 normal lung samples) and plasma (17 lung cancer samples and 19 control samples) of lung cancer patients obtained from the GEO database (accession number GSE51853 and GSE17681). From this analysis, we found that the miR-101-3p expression levels were downregulated in both the tissue and plasma of lung cancer patients as opposed to USP47 expression (Figure 7b,c). In addition, lung cancer patients with low miR-101-3p expression exhibited a significantly lower survival rate than those with high miR-101-3p expression (Figure 7d). Conversely, patients with high USP47 expression had a poorer survival rate than those with low USP47 expression (Figure 7e). Next, we tested the expression pattern of miR-101-3p in various lung cancer cell lines, including A549, H157, H358, H1299, and H1703, as in the results obtained from the GEO database. The qRT-PCR results showed that the miR-101-3p levels were downregulated in several lung cancer cell lines relative to a normal lung cell (L132 cell) (Figure S7). These results suggest that abnormal expressions of miR-101-3p and USP47 are associated with tumorigenesis and a poor prognosis in clinical patients with lung cancer.

## 4. Discussion

MiR-101-3p has been found to participate in multiple cancer-related biological processes, including proliferation, apoptosis, invasion, and metastasis. In particular, it serves as a tumor suppressor in diverse malignancies by targeting critical oncogenes or anti-oncogenes, including EZH2 [21], DNMT3a [19], MALAT-1 [20], ZEB1 [23], and USP47 [24]. Levin and his colleagues showed that miR-101-3p suppresses cancer progression via p53 accumulation by targeting the proteasome maturation protein (POMP), which is involved in the biogenesis of proteasomes [22]. In addition, Fujiwara et al. suggested a positive feedback loop involving miR-101-3p and p53 mediated by nucleolar stress [12]. Thus, miR-101-3p appears to regulate tumorigenesis in a p53-dependent manner.

In this study, we showed that a reduction in USP47 expression by miR-101-3p increases RPL11 ubiquitination and translocation to the nucleus, thereby promoting the interaction between RPL11 and MDM2, and consequently protecting p53 protein degradation. USP47 is a deubiquitinating enzyme reported as an oncogene [29,30]. It binds to β-TRCP and regulates the levels of its substrate, Cdc25A. A depletion in USP47 decreases cell survival by inducing the accumulation of Cdc25A, and has anti-cancer effects on several cancer cell lines [31]. Furthermore, it has been reported that miR-204-5p reduces USP47 levels by directly targeting it and inhibiting cell proliferation in gastric cancer [32]. Previously, we reported that a loss of USP47 inhibits cancer cell growth by increasing p53 stability and activity [25]. 

Based on these evidences, we speculated that USP47 levels regulated by miR-101-3p may be involved in the accumulation of p53 in cancer cells. We proved this hypothesis by showing that the miR-101-3p-mediated reduction in p53 levels was restored by USP47, and that the inhibition of cell proliferation by miR-101-3p was also rescued by the overexpression of USP47 in p53-positive cancer cells. A recently published paper demonstrated that long-non-coding RNA (lncRNA) DSCAM-AS1 play a role as a sponge for miR-101-3p, resulting in the exacerbation of osteosarcoma progression by protecting the miR-101-3p-mediated depletion of USP47 [24]. However, the paper did not show whether the activity of miR-101-3p is also prevented by DSCAM-AS1 in their models. To solve this question, we will investigate whether this lncRNA affects the interaction of USP47 with RPL11 or the deubiquitination and translocation of RPL11 by USP47, and thus is involved in the p53 protein levels as in our models.

Ribosomal biogenesis is a complex cellular process that consumes substantial amounts of energy. Defects in ribosomal biogenesis cause ribosomal stress, signaling a pathway that involves the inhibition of MDM2 E3-ligase activity by several ribosomal proteins and p53 activation. The complex of 5S rRNA, RPL5, and RPL11 is the main regulator of ribosomal stress signaling. Upon ribosomal stress, RPL11 inhibits MDM2 activity and protects p53 from MDM2-mediated proteasomal degradation [14,16,33]. Indeed, since ribosomal stress induces the post-transcriptional activation of miR-101-3p [12], we believe that the overexpression of the miR-101-3p mimic has the same effect as the upregulation of miR-101-3p due to ribosomal stress. The overexpression of the miR-101-3p mimic increased the interaction between RPL11 and MDM2 and the accumulation of p53 as under ribosomal stress conditions. Additionally, it has already been reported that USP47 is released from RPS2 in response to ribosomal stress, and therefore ubiquitinated RPS2 inhibits MDM2 and activates p53 [25]. Similar to RPS2, when ribosomal stress occurs, the reduction in USP47 by miR-101-3p leads to an accumulation of ubiquitinated RPL11 in the nucleoplasm, where RPL11 binds to MDM2, and prevents p53 degradation by MDM2.

Some post-translational modification of RPL11 is required for signaling function to p53. RPL11 is neddylated by MDM2 in the cytoplasm, and the neddylated form of RPL11 is localized at the nucleolus under normal conditions [34]. Ribosomal stress promotes the relocation of RPL11 to the nucleoplasm, resulting in increased interaction with MDM2 and the activation of p53. Furthermore, recent studies have reported that the sumoylation of RPL11 is associated with the translocation of RPL11 to the nucleoplasm during ribosomal stress. The SUMO conjugation of RPL11 is enhanced by ribosomal stress, and the upregulation of this modification inhibits the neddylation of RPL11, resulting in the release of RPL11 from the nucleolus to the nucleoplasm [35]. In this study, we identified that miR-101-3p increased the ubiquitination of RPL11 and the interaction with MDM2 via the translocation of RPL11. These effects of miR-101-3p on RPL11 were eliminated by wild-type USP47, not by catalytically inactive USP47, suggesting that the deubiquitinase activity of USP47 is required for the function of RPL11. From these data, we can speculate that the ubiquitination of RPL11, increased by miR-101-3p, is also critical for RPL11 localization and function in association with p53, and that ubiquitination may be associated with the interplay between neddylation and sumoylation. Further studies are required to evaluate the effects of ubiquitination on the RPL11 function and how these modifications—neddylation, sumoylation, and ubiquitination—could affect each other to regulate RPL11 localization and function during normal or ribosomal stress conditions.

## 5. Conclusions

**Figure 8:**
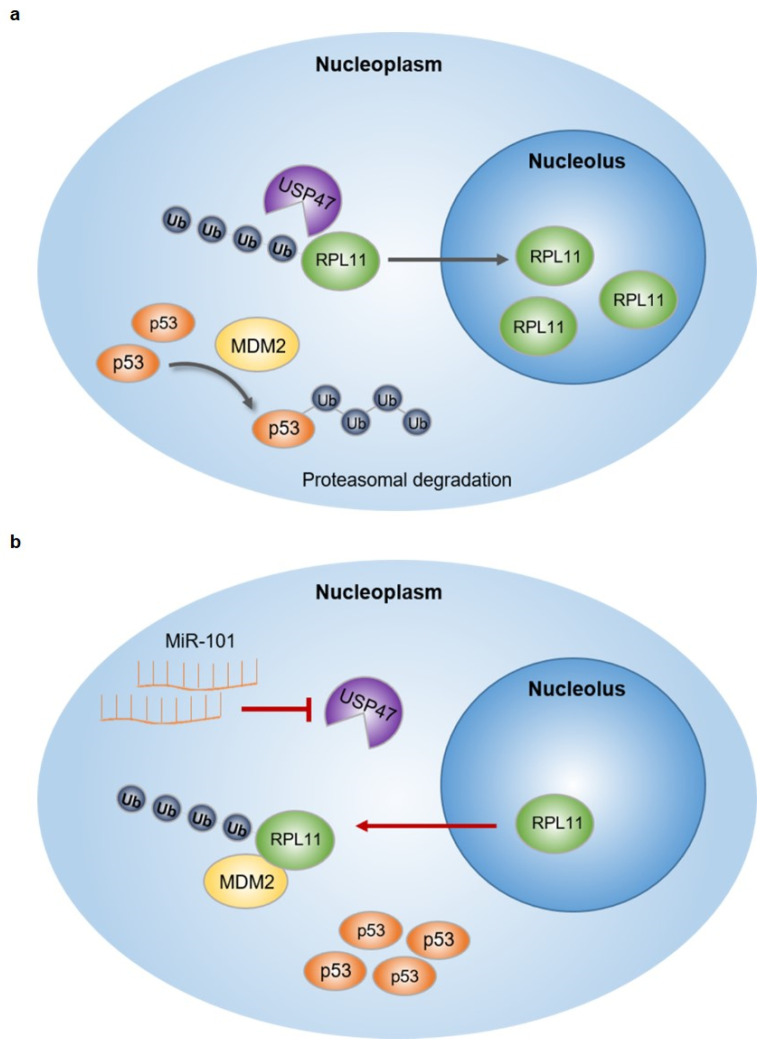
Schematic models for miR-101-3p function in lung cancer cells. (**a**) In cancer cells, increased USP47 deubiquitinates RPL11 and places it in the nucleolus. As a result, free-MDM2 can ubiquitinate p53 and induce its proteasomal degradation, which in turn promotes cell proliferation. (**b**) When miR-101-3p is overexpressed, USP47 levels are reduced by miR-101-3p and the ubiquitinated RPL11 shifts to the nucleoplasm, binds to MDM2, and inhibits p53 degradation by MDM2.

In summary, our study showed that the regulation of RPL11 ubiquitination through a reduction in USP47 levels by miR-101-3p affects RPL11 localization and function, resulting in the activation of p53 and the inhibition of cell proliferation in cancer cells (Figure 8a,b). Thus, we provided an innovative evidence for miR-101-3p to function as a tumor suppressor, and powerful evidence of the possibility of miR-101-3p as a potential therapeutic target for cancers.

## Data Availability

The data presented in this study are contained within the article or Supplementary Materials.

## Supplementary Materials

The following are available online at https://www.mdpi.com/article/10.3390/cancers14040964/s1, File S1: Original images of the western blot, Figure S1: Transfection efficiency of miR-101-3p mimic into the cells, Figure S2: The function of miR-101-3p dependent on p53, Figure S3: MiR-101-3p directly targets the *USP47* gene, Figure S4: MiR-101-3p inhibits cell proliferation by targeting USP47 in H1650 cells, Figure S5: p53 protein stability is maintained by miR-101-3p, Figure S6: RPL11 ubiquitination is reduced by USP47 knockdown, Figure S7: The abundance of miR-101-3p in lung cancer cell lines.
