# Supplementary material for: MicroRNA-101-3p Suppresses Cancer Cell Growth by Inhibiting the USP47-Induced Deubiquitination of RPL11
Source: Cancers (Basel). 2022 Feb 15;14(4):964. doi: 10.3390/cancers14040964 (PMC8870143; doi:10.3390/cancers14040964)
Supplement: Supplementary file 1 [file cancers-14-00964-s001.zip › cancers-1585777-supplementary-File S1.pdf]

Fig. 1e

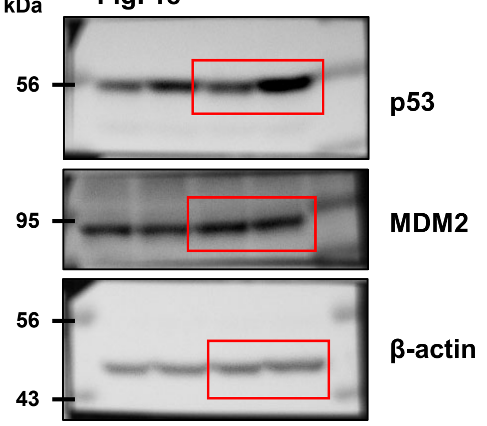

Fig. 1f

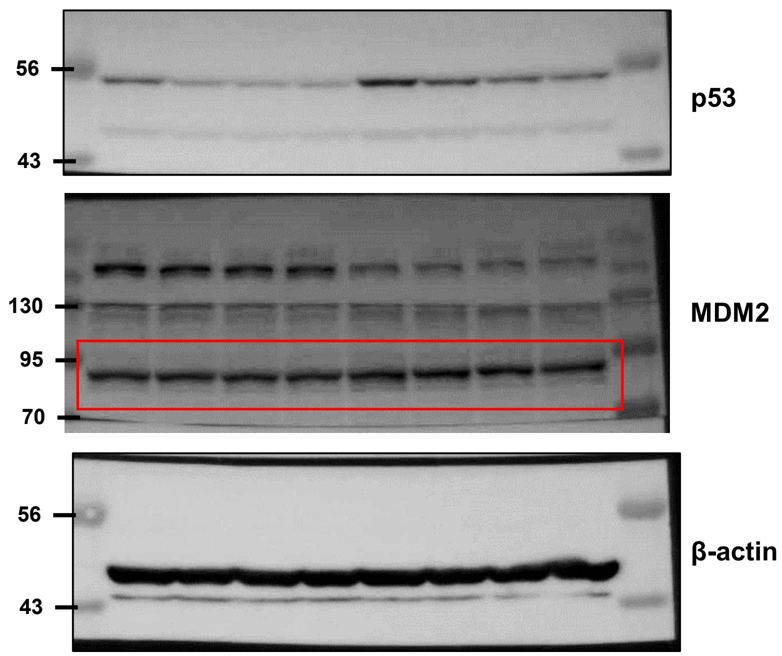

Fig. 2a

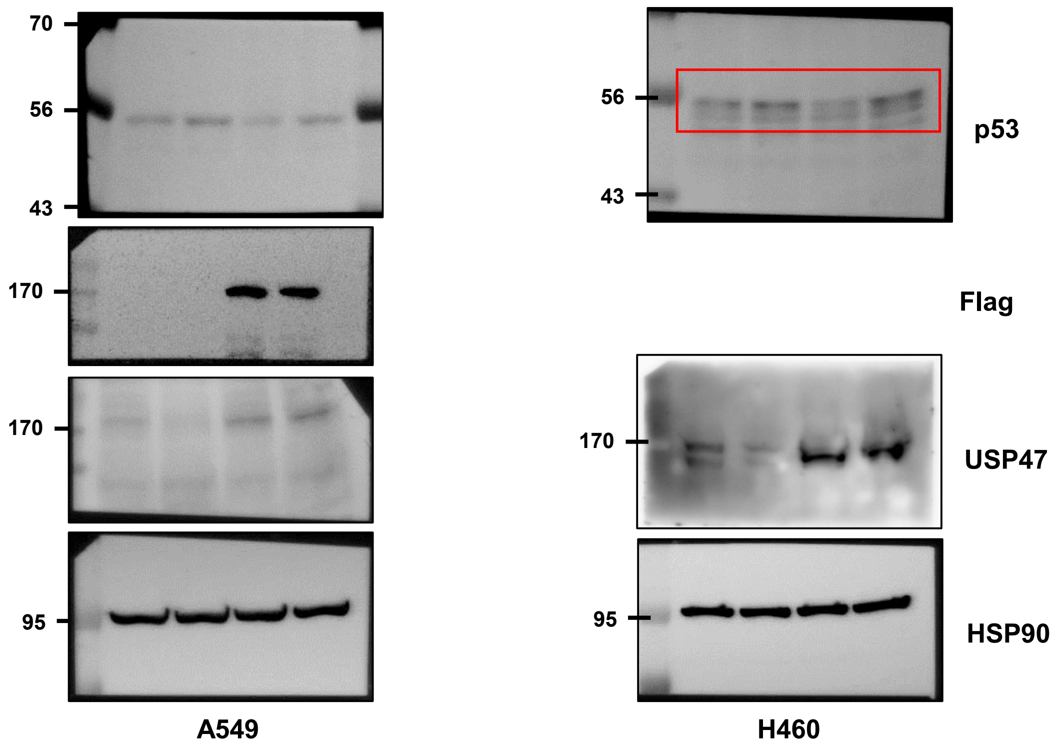

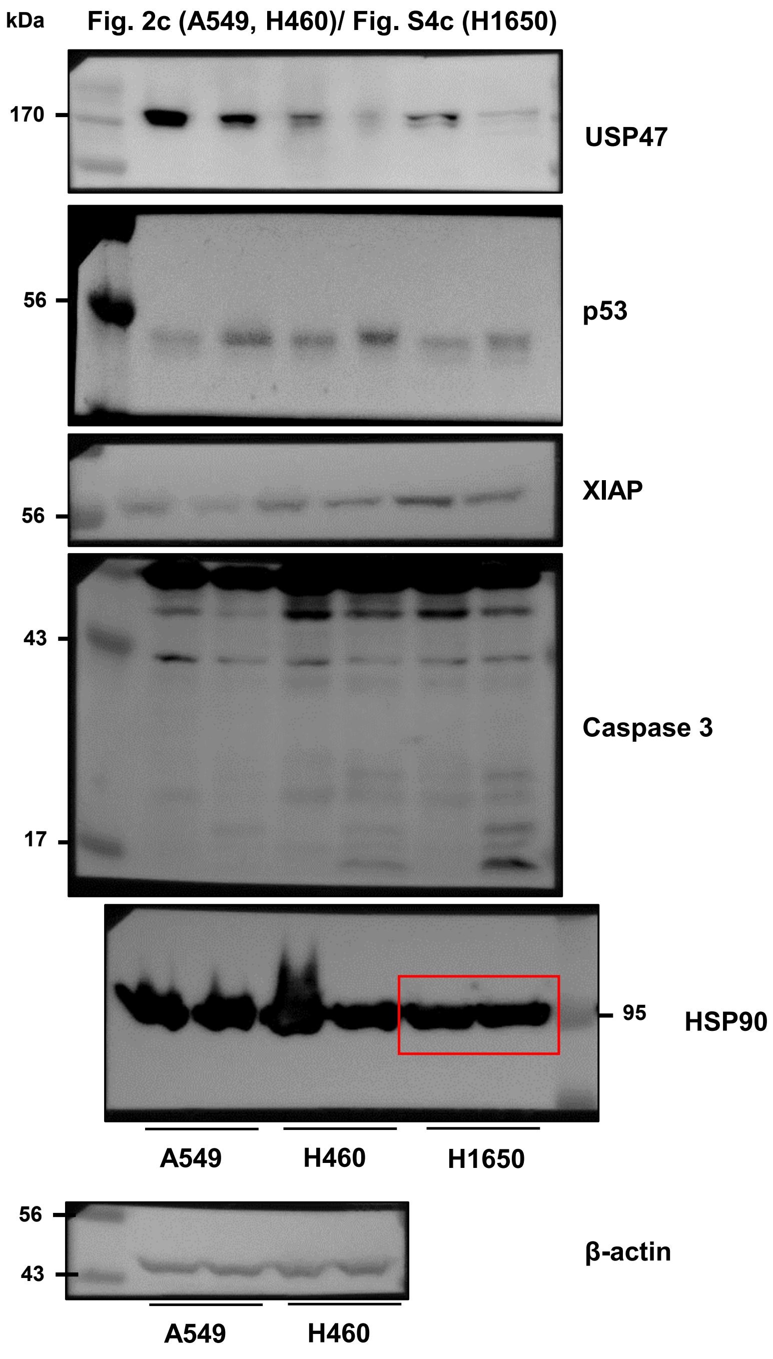
kDa
Fig. 2c (A549, H460)/ Fig. S4c (H1650)
170
USP47
56
p53
XIAP
56
43
Caspase 3
17
95
HSP90
A549
H460
H1650
56
43
β-actin
A549
H460

Fig. 4a

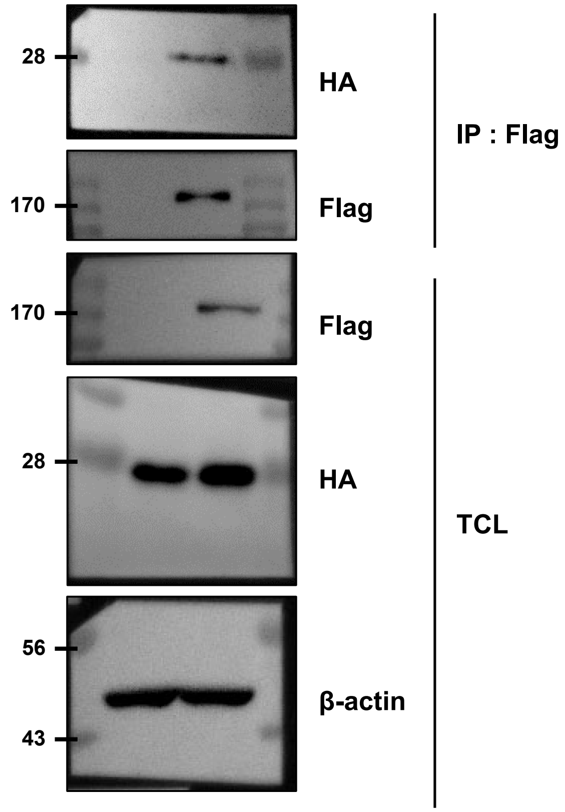
kDa
28
HA
170
Flag
IP : Flag
170
Flag
28
HA
TCL
56
β-actin
43

Fig. 4b

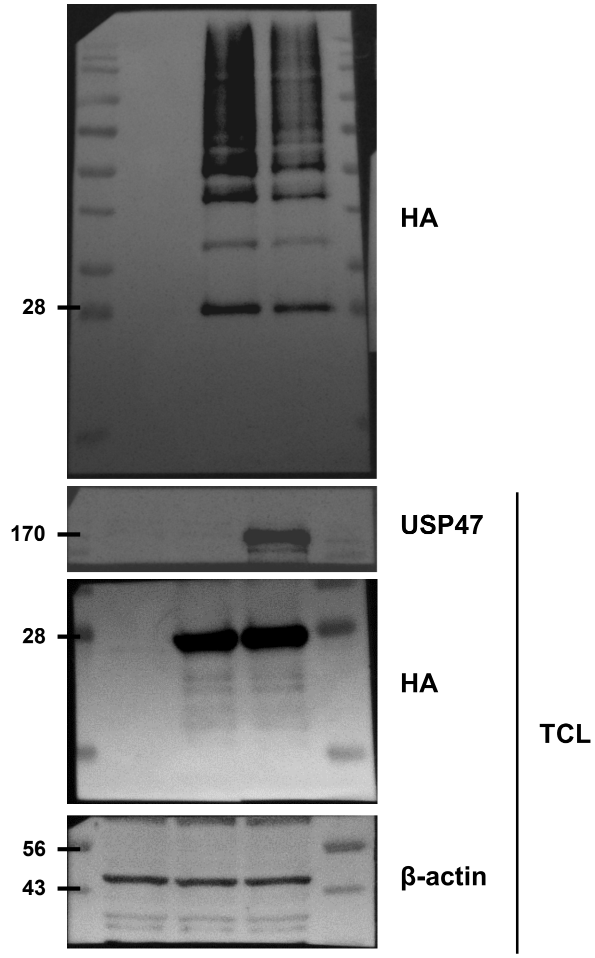
kDa
HA
28
170
USP47
28
HA
TCL
56
β-actin
43

Fig. 4c

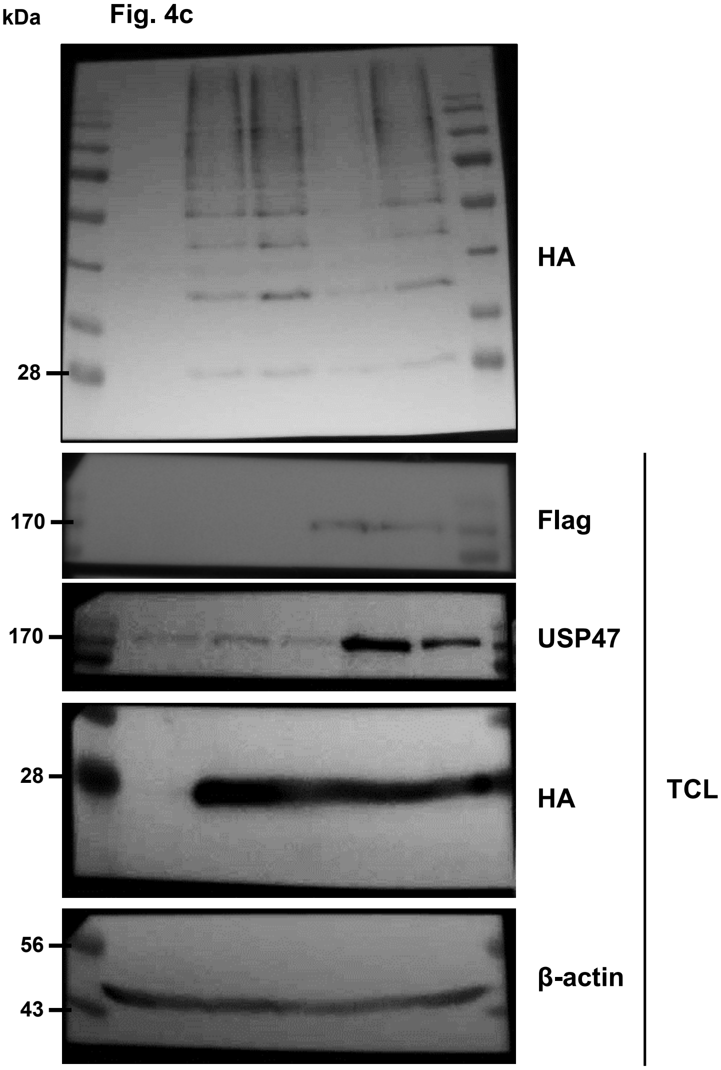
kDa
28
HA
170
Flag
170
USP47
28
HA
TCL
56
43
β-actin

Fig. 4d

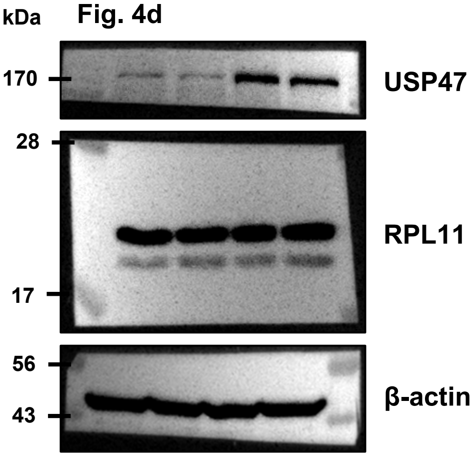
kDa
170
USP47
28
RPL11
17
56
43
β-actin

Fig. 5a

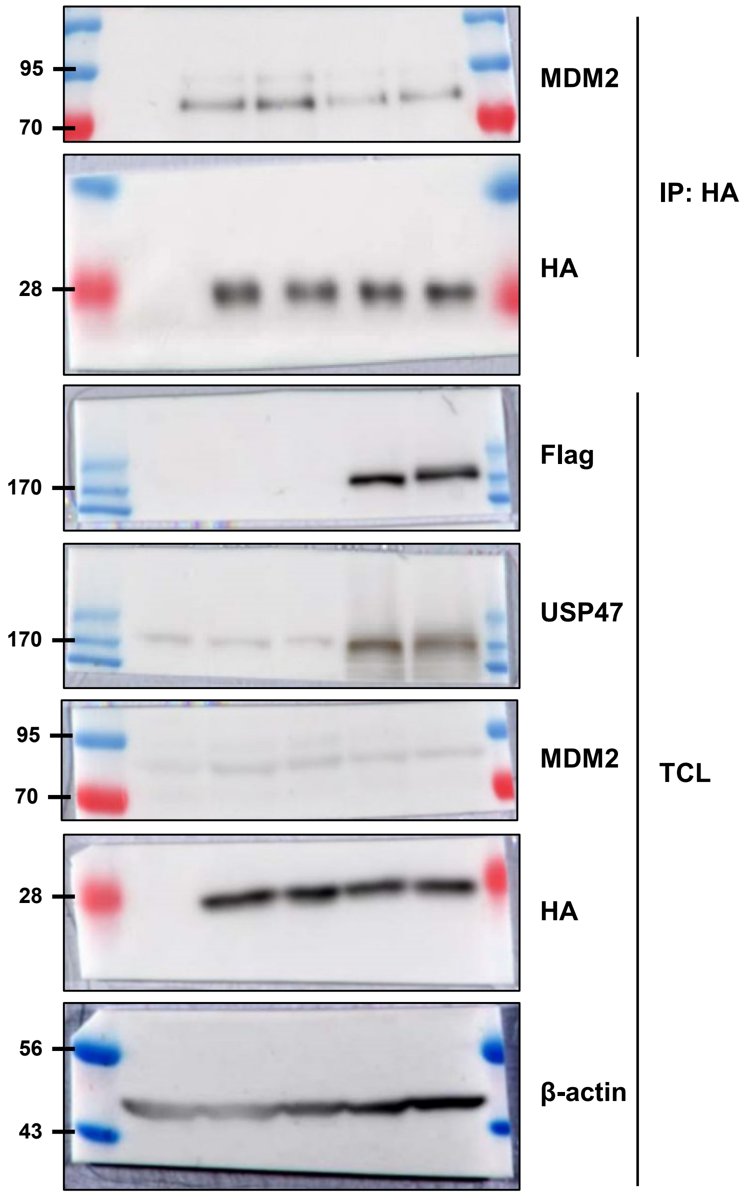
kDa
95
70
MDM2
28
HA
IP: HA
170
Flag
170
USP47
95
70
MDM2
TCL
28
HA
56
43
β-actin

Fig. 5c

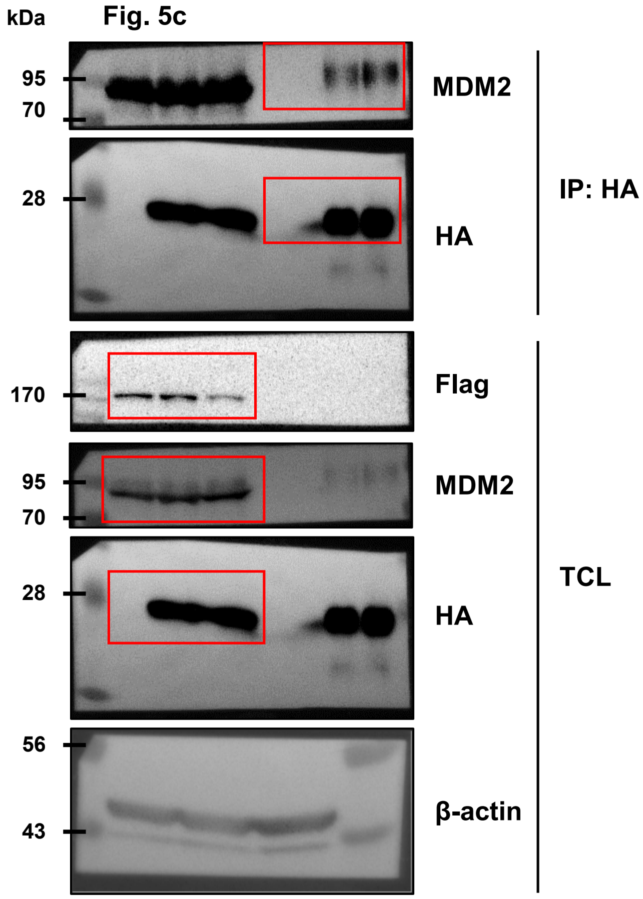
kDa
95
70
MDM2
28
HA
IP: HA
170
Flag
95
70
MDM2
TCL
28
HA
56
43
β-actin

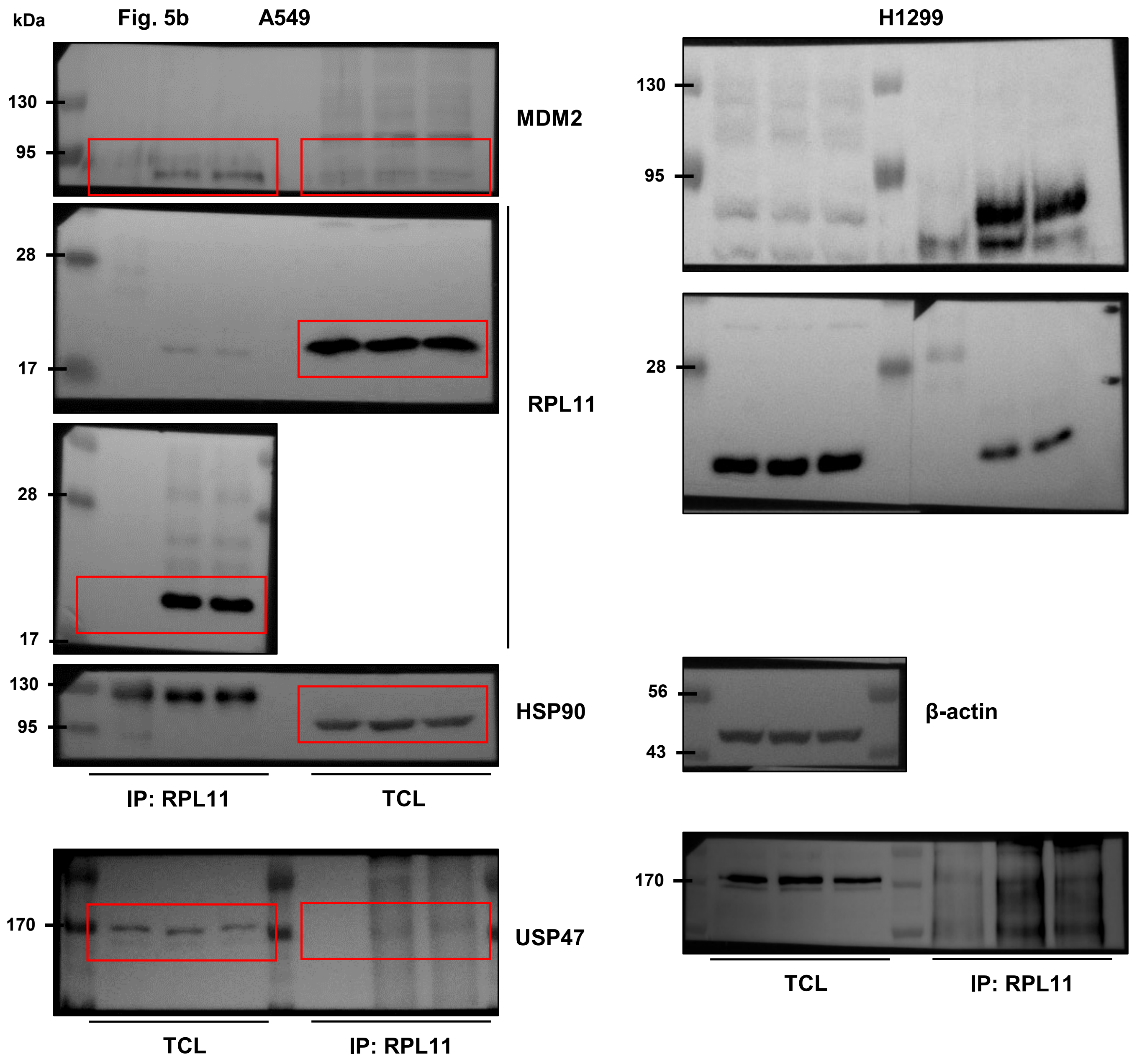
kDa
Fig. 5b
A549
H1299
130
95
MDM2
28
17
RPL11
28
17
130
95
HSP90
IP: RPL11
TCL
170
USP47
TCL
IP: RPL11
130
95
28
56
43
β-actin
170
TCL
IP: RPL11

Fig. 6c

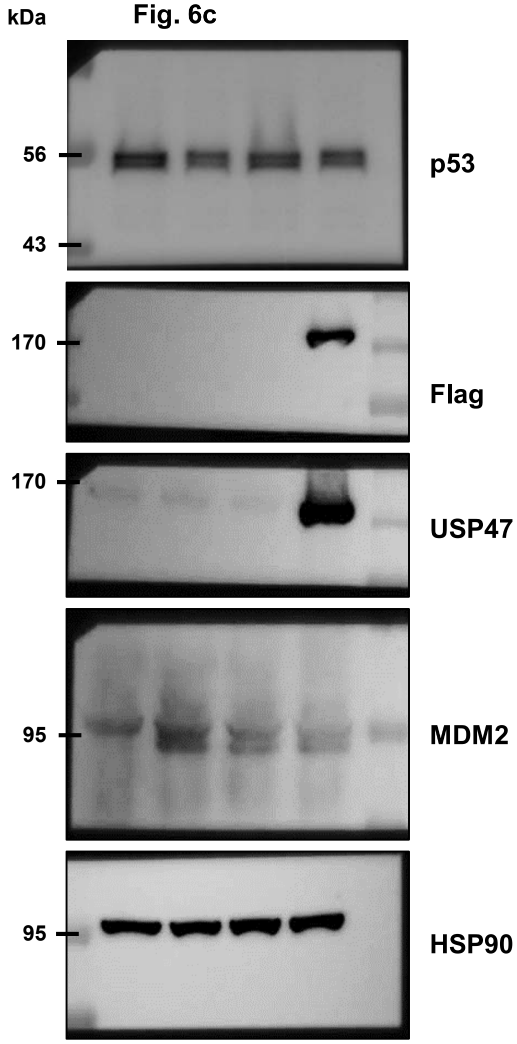

Fig. S3b

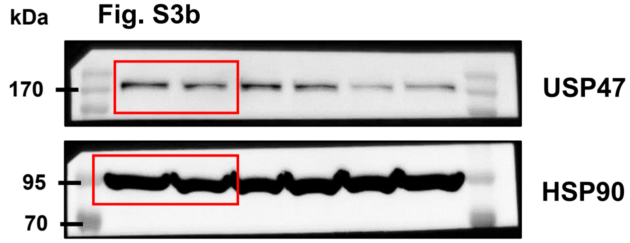

Fig. S4a

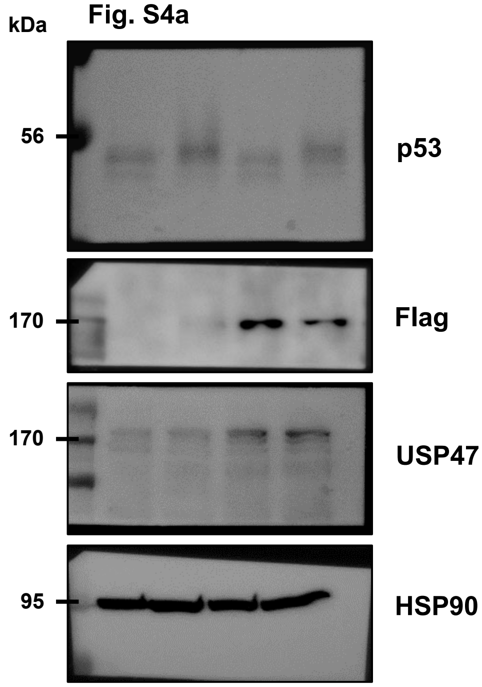

Fig. S5

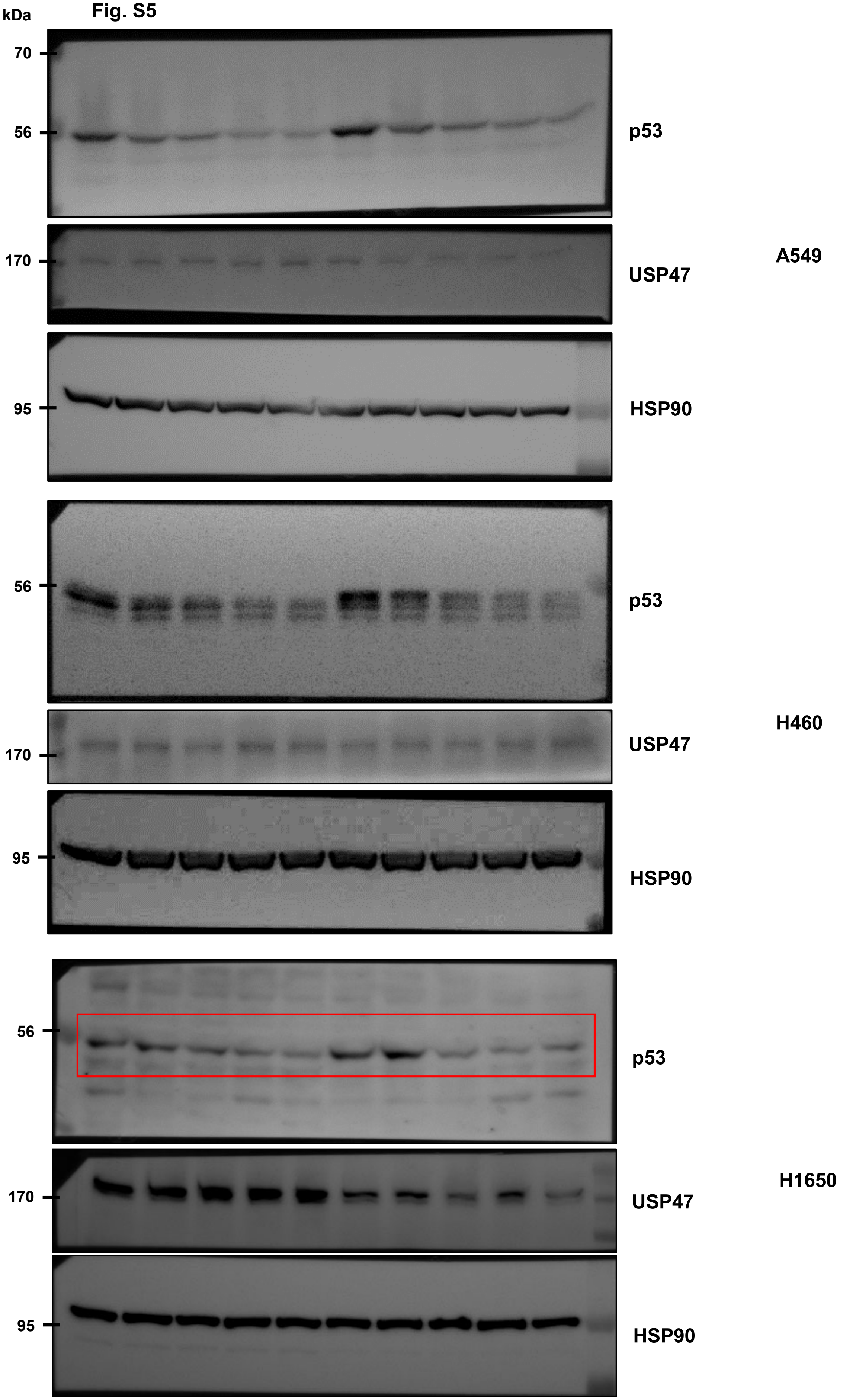
kDa
70
56
p53
170
USP47
A549
95
HSP90
56
p53
170
USP47
H460
95
HSP90
56
p53
170
USP47
H1650
95
HSP90

kDa Fig. S6a

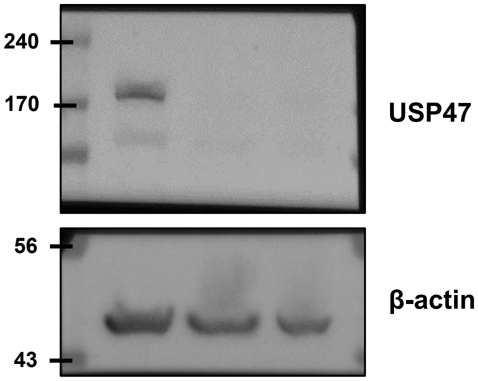

kDa Fig. S6b, A549

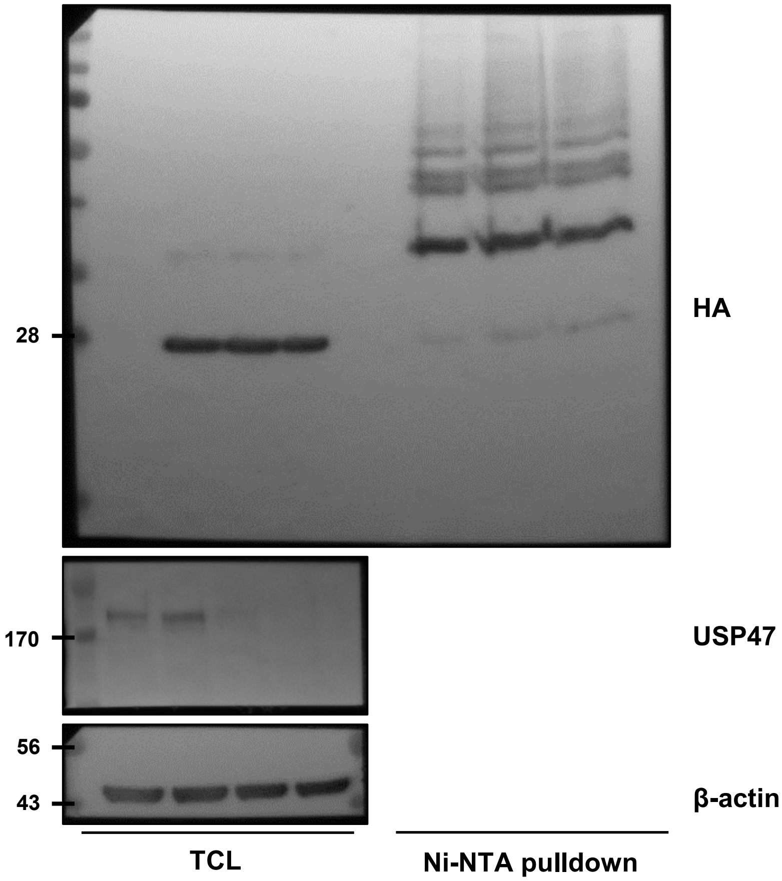

kDa Fig. S6b, H460

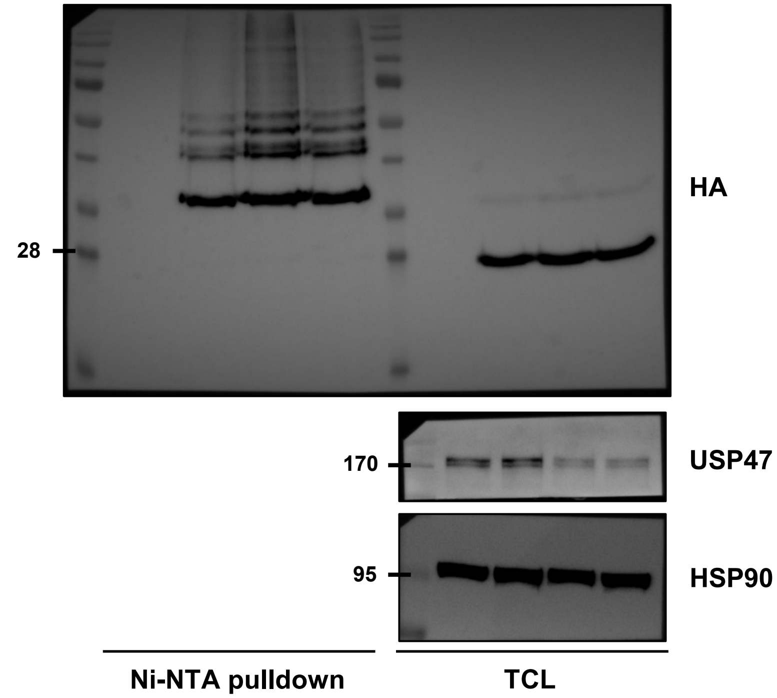

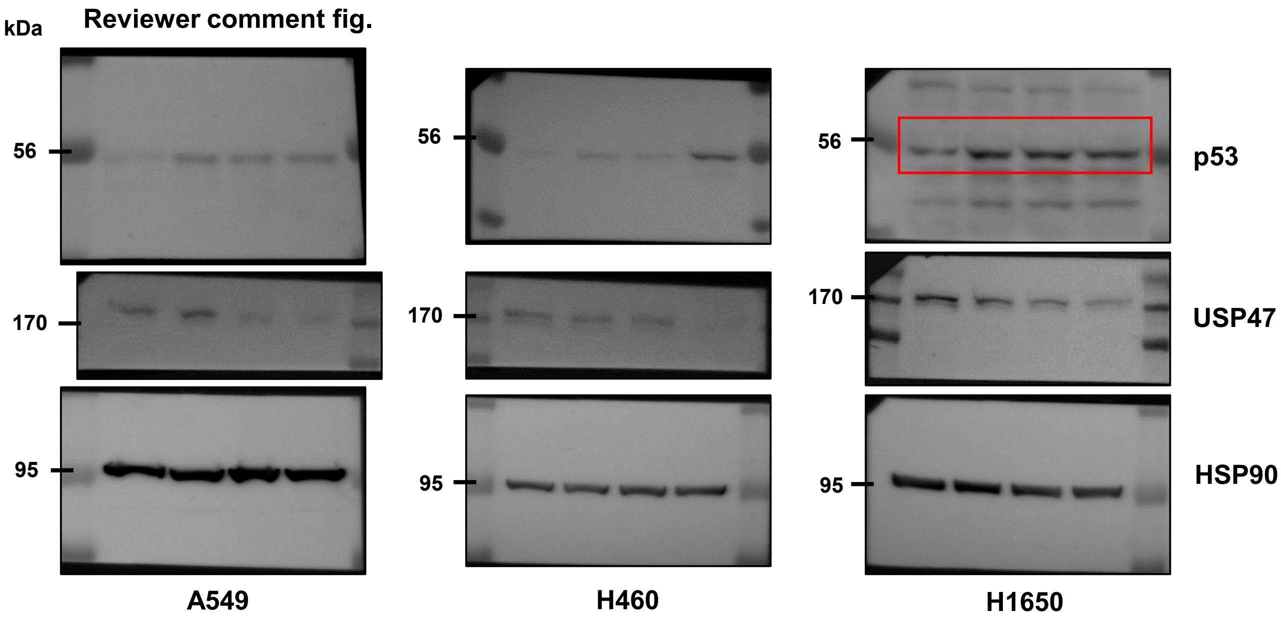
kDa
Reviewer comment fig.
56
170
95
56
170
95
56
170
95
p53
USP47
HSP90
A549
H460
H1650
