# Supplementary material for: MicroRNA-101-3p Suppresses Cancer Cell Growth by Inhibiting the USP47-Induced Deubiquitination of RPL11
Source: Cancers (Basel). 2022 Feb 15;14(4):964. doi: 10.3390/cancers14040964 (PMC8870143; doi:10.3390/cancers14040964)
Supplement: Supplementary file 1 [file cancers-14-00964-s001.zip › cancers-1585777-supplementary.pdf]

Supplementary Materials

MicroRNA-101-3p Suppresses Cancer Cell Growth by Inhibiting the USP47-Induced Deubiquitination of RPL11

Jinyoung Park, Moonsoo Cho, Jinhong Cho, Eunice EunKyeong Kim and Eun Joo Song

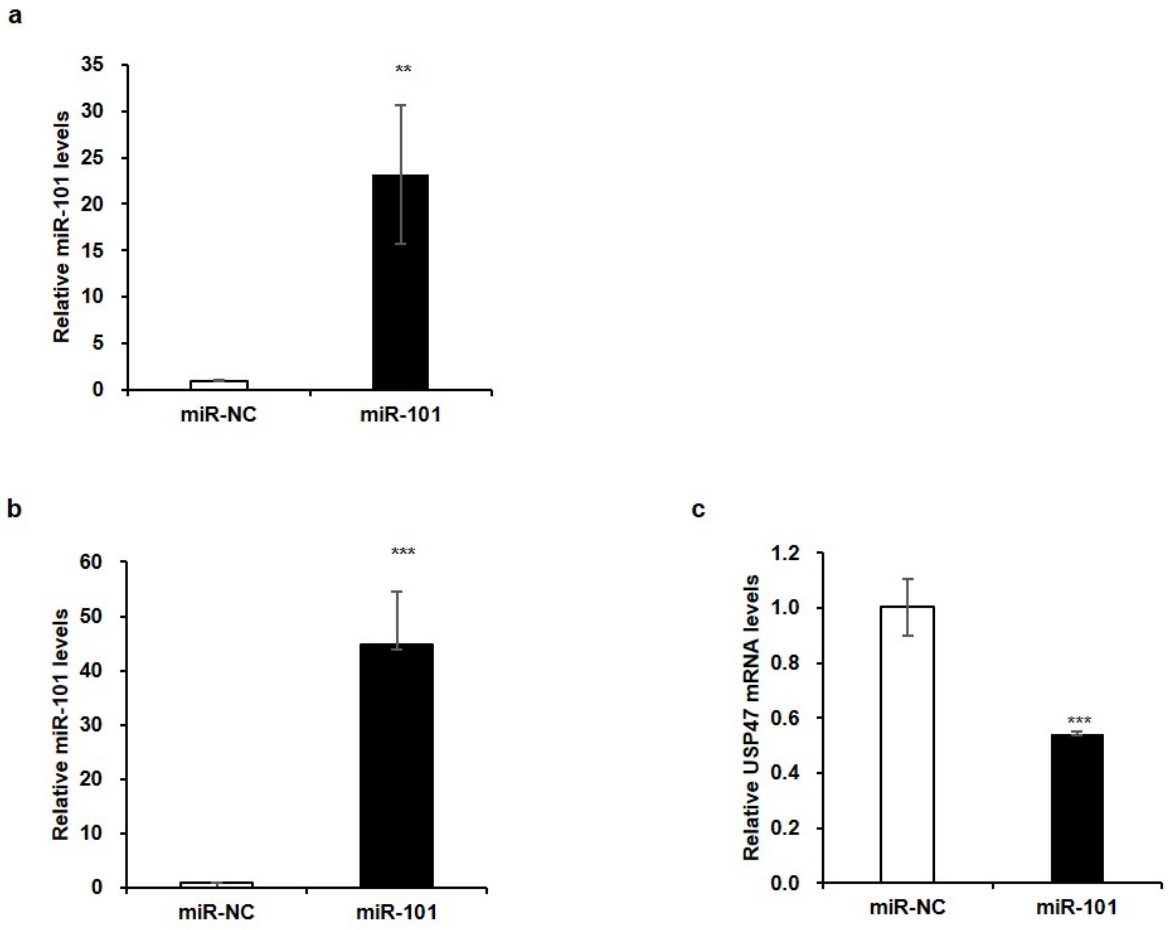

Figure S1. Transfection efficiency of miR-101-3p mimic into the cells. (**a**) A549 cells were transfected with or without miR-101-3p mimic. Q-PCR analysis was conducted to determine the relative expression levels of miR-101. (**b**,**c**) HEK293T cells were transfected with or without miR-101-3p mimic. Q-PCR analysis was conducted to determine the relative expression levels of (**b**) miR-101 or (**c**) USP47 mRNA. The data are shown as mean ± SD and determined by three independent repeated experiments (** $p < 0.005$, *** $p < 0.001$, Student's *t*-test).

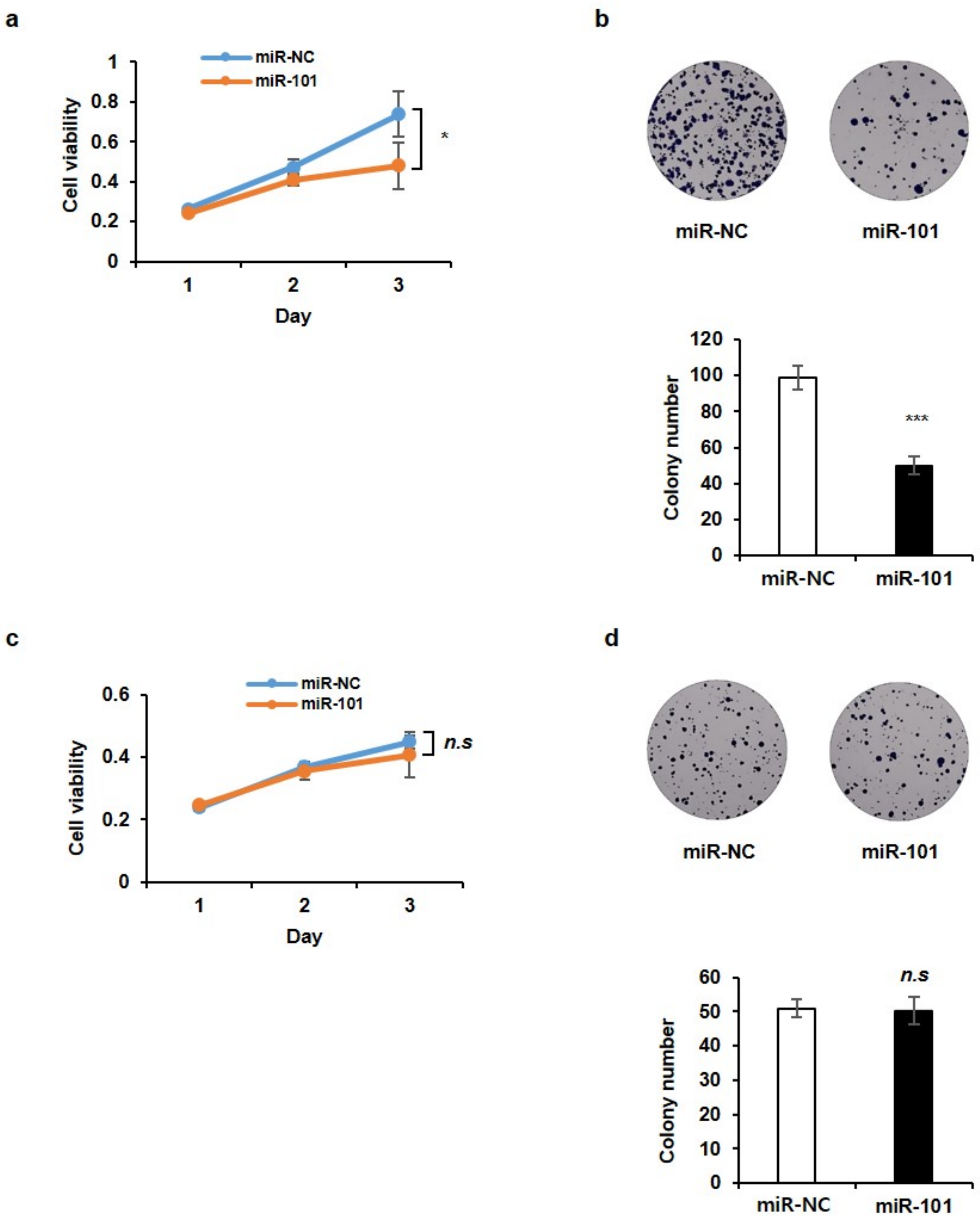

Figure S2. The function of miR-101-3p dependent on p53. (**a**,**b**) Using HCT116 (p53+/+) cells transfected with miR-101-3p mimic, cancer cell growth was measured at the time indicated by (**a**) the WST-1 assay or (**b**) colony formation assay. The number of colonies for each group was normalized to the control. (**c**,**d**) Using HCT116 (p53−/−) cells, cancer cell growth was measured at the time indicated by (**c**) the WST-1 assay or (**d**) colony formation assay. The number of colonies for each group was normalized to the control. The data are shown as mean ± SD and determined by three independent repeated experiments (* $p < 0.05$, *** $p < 0.001$, Student's *t*-test). *n.s*, non-specific.

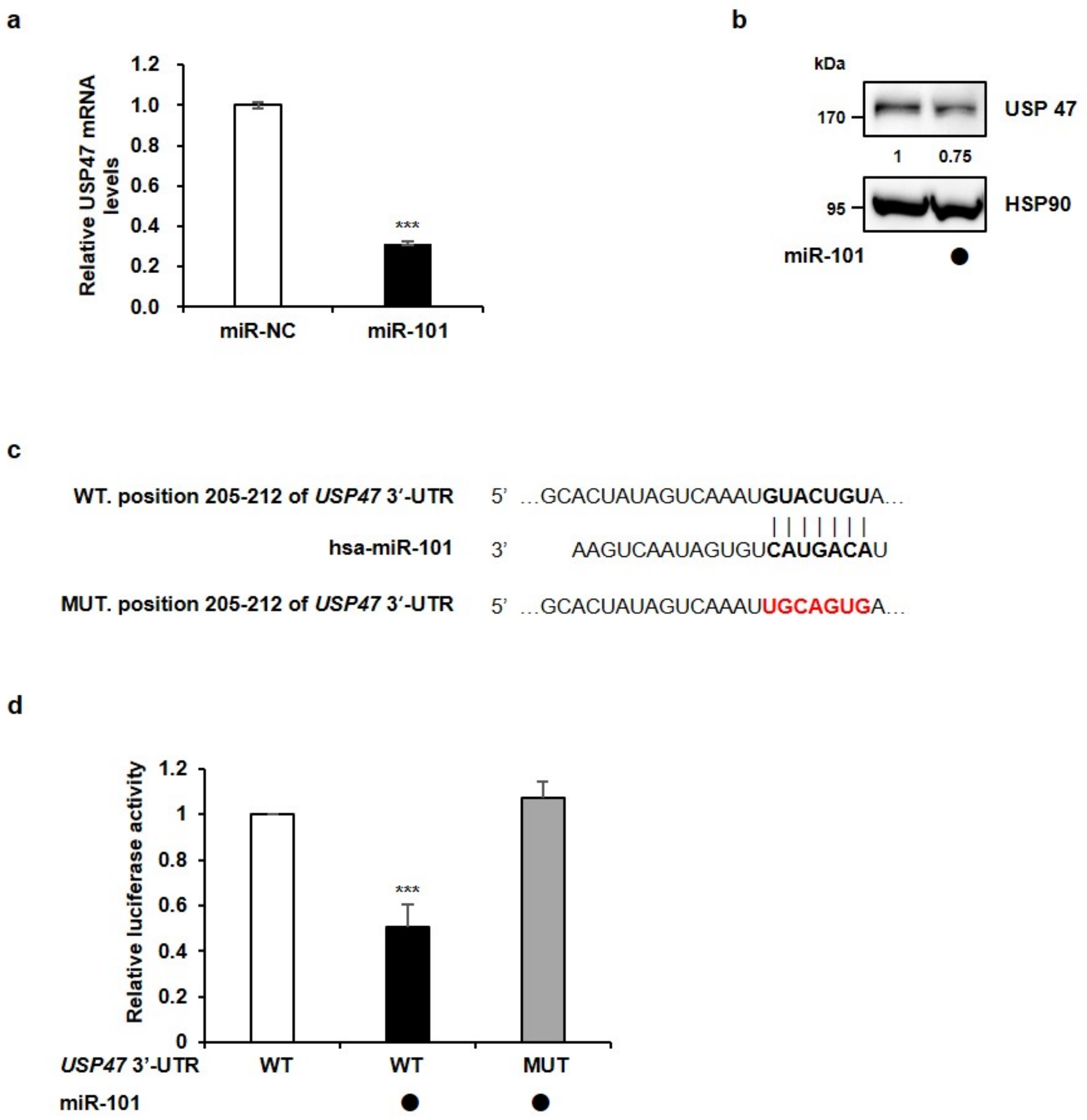

Figure S3. MiR-101-3p directly targets the *USP47* gene. (**a**) A549 cells were transfected with or without miR-101 mimic. Analysis was conducted to determine the relative expression levels of mRNAs encoding USP47. (**b**) Western blot analysis was performed to detect USP47. (**c**) A schematic of the predicted miR-101-3p binding site in the 3'-UTR region of *USP47* mRNA. Wild-type sequences and mutant sequences of the binding site in the 3′-UTR regions of *USP47* and miR-101 sequences are shown. (**d**) A549 cells were transfected with pmiR-GLO vectors that expressed the luciferase reporter gene under the control of either the wild-type *USP47* 3′-UTR or a mutant *USP47* 3′-UTR, either alone or in combination with miR-101 mimic. Luciferase activity was measured 48 h after transfection, and firefly luciferase activity was normalized to renilla luciferase activity. Data are shown as mean ± SD (*** $p < 0.001$, *t*-test) and determined by three independent repeated experiments.

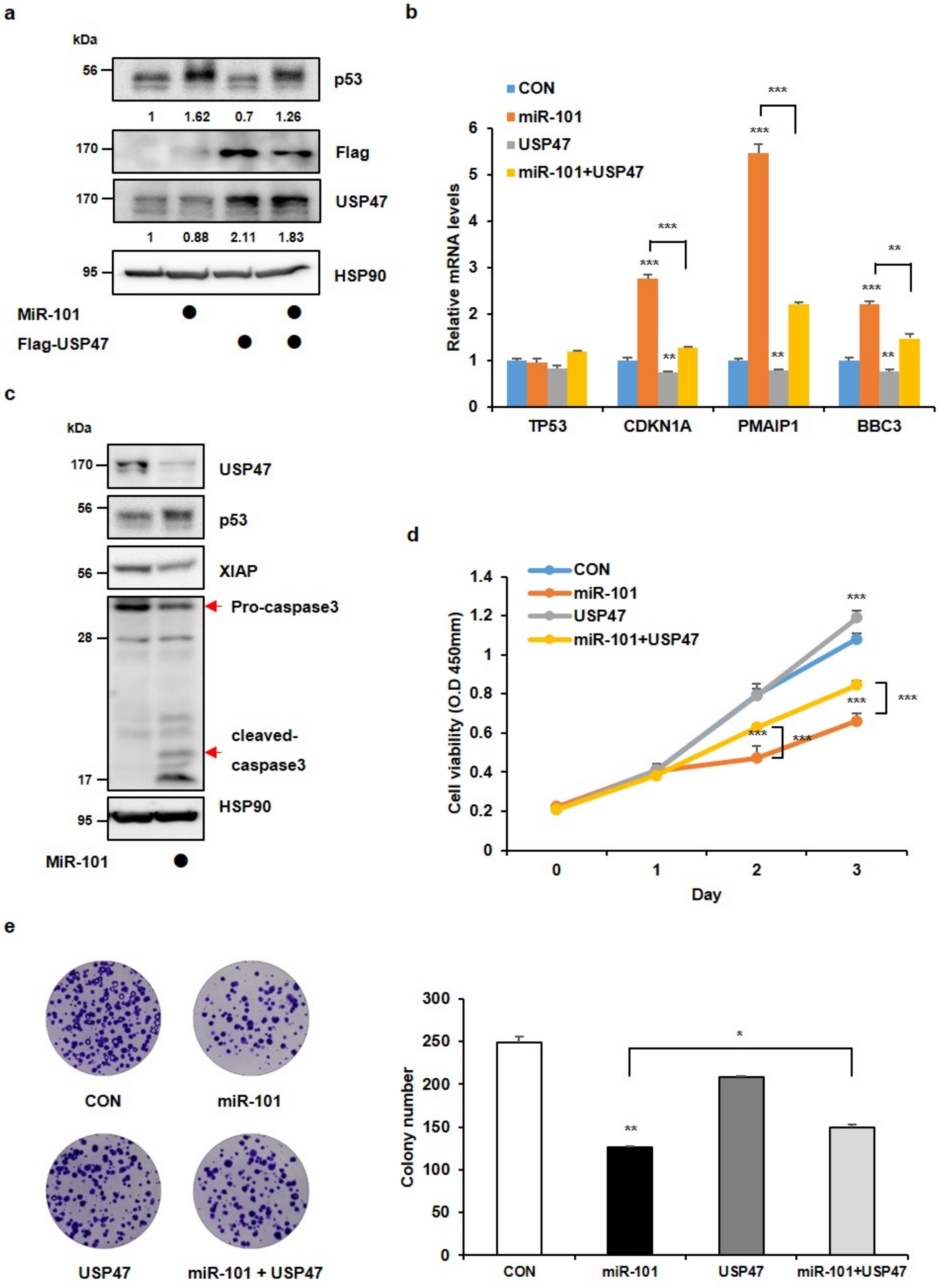

Figure S4. MiR-101-3p inhibits cell proliferation by targeting USP47 in H1650 cells. (**a**,**b**) H1650 cells were transfected with miR-101-3p mimic or Flag-USP47 alone, or together both. (**a**) Western blot analysis was performed to detect the p53 and USP47 protein levels. (**b**) Q-PCR analysis was conducted to determine the relative expression levels of p53 and its target genes. (**c**) H1650 cells were transfected with miR-101-3p mimic. Western blot analysis was per-formed to detect apoptotic proteins. (**d**,**e**) H1650 cells were transfected with miR-101-3p mimic or Flag-USP47 alone, or together both. (**d**) Cell viability was measured at the time indicated by the WST-1 assay. (**e**) 15 days after transfection, cells were stained with crystal violet

in a 35 mm dish. The number of colonies for each group was normalized to the control (CON). Data are shown as mean ± SD (* $p < 0.05$, ** $p < 0.005$, *** $p < 0.0005$, *t*-test) and determined by three independent repeated experiments.

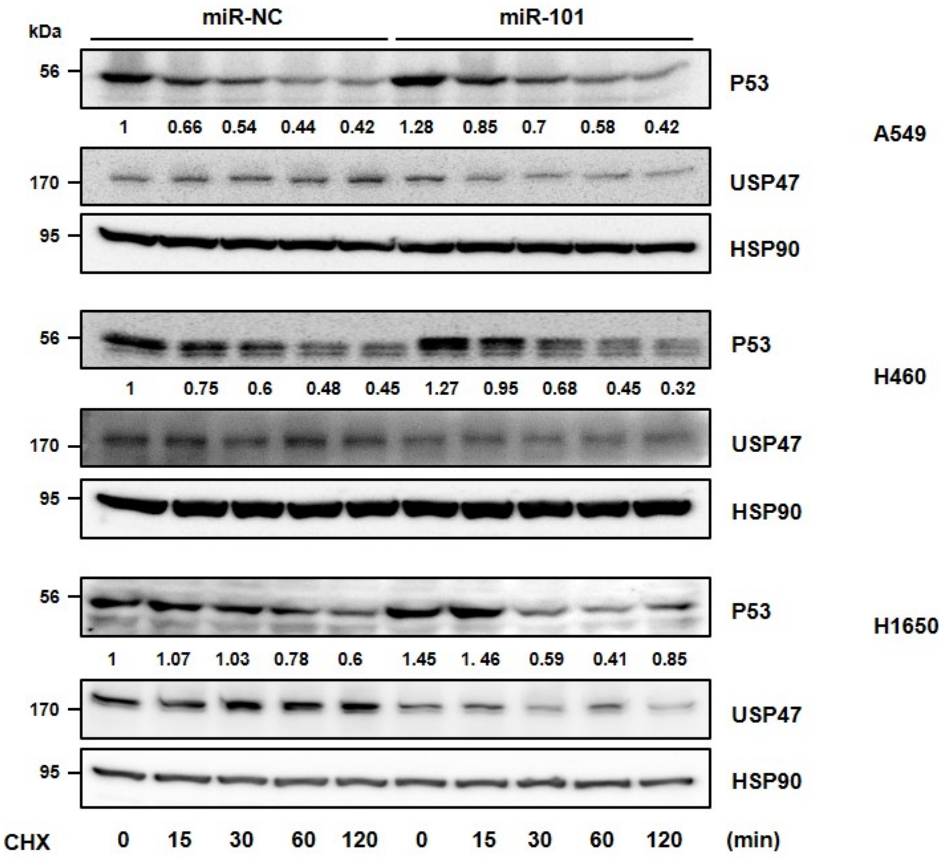

Figure S5. p53 protein stability is maintained by miR-101-3p. A549, H460, and H1650 cells were transfected with miR-101-3p mimic. After transfection, cells were treated with 100 μg/mL CHX and harvested indicated time. Western blot analysis was performed to detect USP47 and p53 proteins.

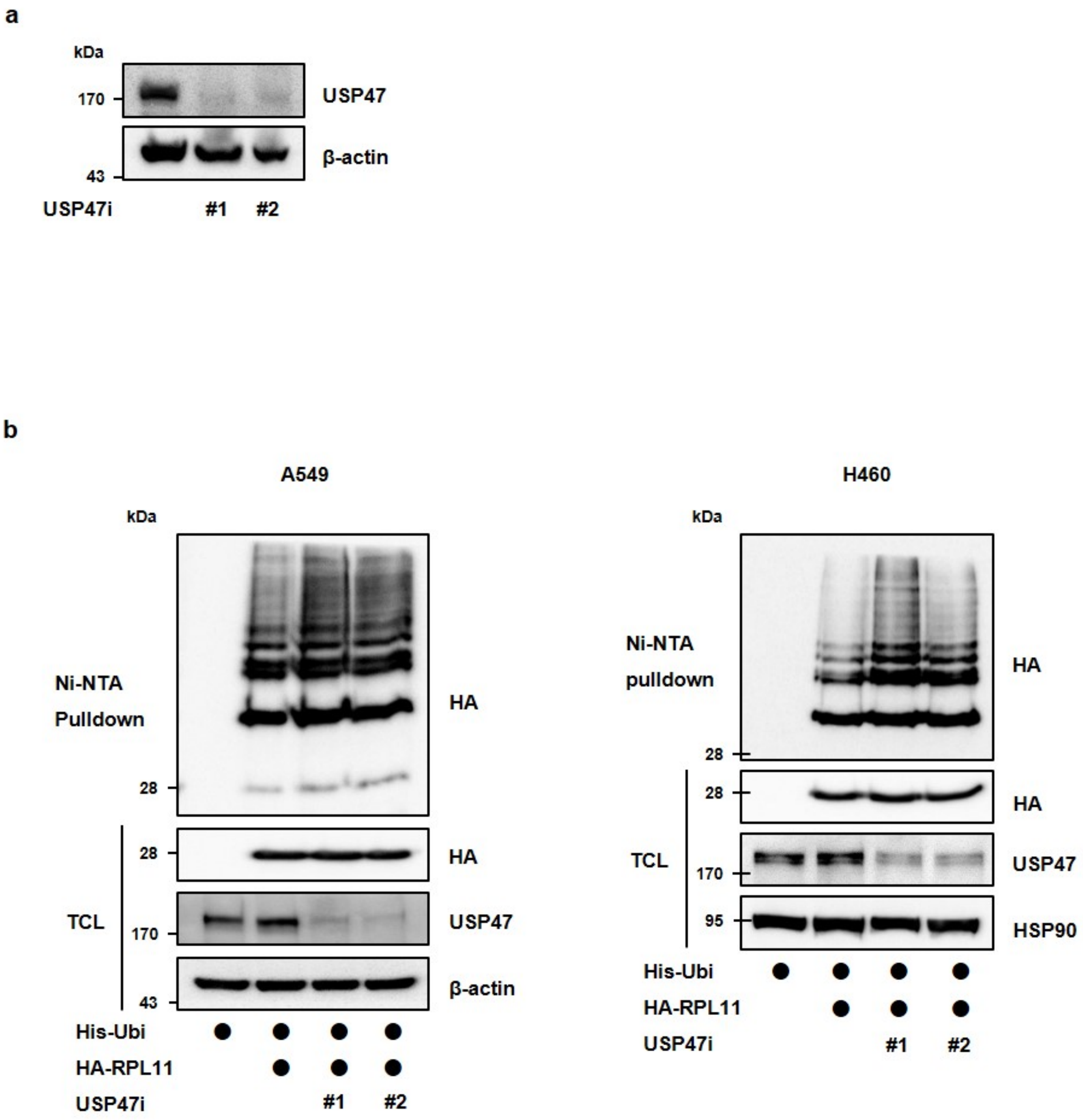

Figure S6. RPL11 ubiquitination is reduced by USP47 knockdown. (**a**) HEK293T cells were transfected with siRNAs targeting *USP47* (USP47i - #1, #2). Western blot analysis was performed to detect USP47. (**b**) A549 and H460 cells were transfected with His-ubiquitin alone or together with HA-RPL11 and USP47i - #1, #2 and then treated with proteasomal inhibitor MG132 (10 μM) for 4 h. RPL11 ubiquitination was observed using the Ni-NTA pulldown assay.

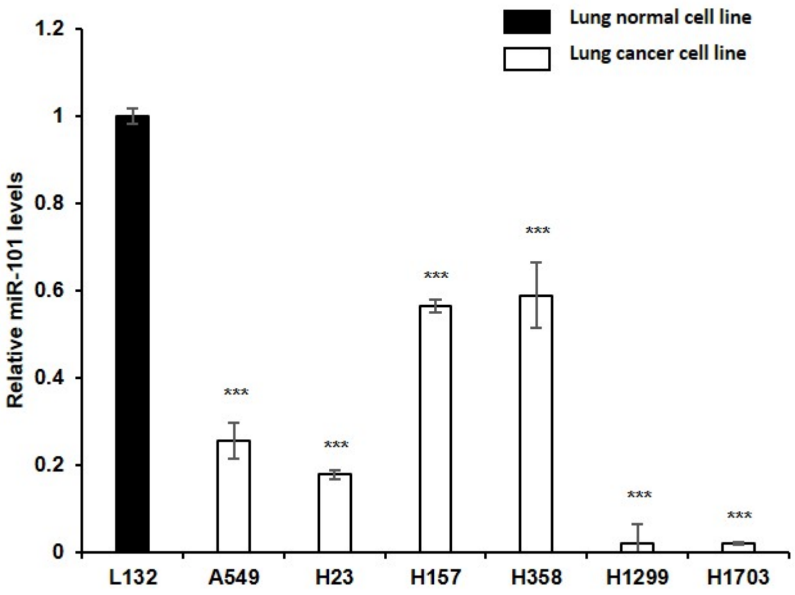

Figure S7. The abundance of miR-101-3p in lung cancer cell lines. Expression levels of miR-101-3p were measured by qRT-PCR in A549, H23, H157, H358, H1299, H1703 or L132 cell lines. The data are shown as mean ± SD of three independent experiments (*** $p < 0.001$, Student's *t*-test).
